# *TP53* ‐Mutated Myeloid Neoplasms: 2024 Update on Diagnosis, Risk‐Stratification, and Management

**DOI:** 10.1002/ajh.27655

**Published:** 2025-03-11

**Authors:** Mithun Vinod Shah, Daniel A. Arber, Devendra K. Hiwase

**Affiliations:** ^1^ Division of Hematology Mayo Clinic Rochester Minnesota USA; ^2^ Department of Pathology University of Chicago Chicago Illinois USA; ^3^ Department of Haematology, Royal Adelaide Hospital Central Adelaide Local Health Network Adelaide South Australia Australia; ^4^ Precision Medicine Theme South Australian Health and Medical Research Institute (SAHMRI) Adelaide South Australia Australia; ^5^ Adelaide Medical School University of Adelaide Adelaide South Australia Australia

**Keywords:** AML—molecular diagnosis & therapy, myelodysplastic syndrome, neoplasia—myeloid leukemias and dysplasias, p53

## Abstract

Alterations in the tumor suppressor gene *TP53* are common in human cancers and are associated with an aggressive nature. Approximately 8%–12% of myelodysplastic syndrome (MDS) and acute myeloid leukemia (AML) harbor *TP53* mutations (*TP53* ^mut^) and present immense challenges due to inherent chemoresistance and poor outcomes. As *TP53* ^mut^ are more common in older individuals and those with secondary/therapy‐related myeloid neoplasms (MN), their incidence is expected to increase with an aging population and rising proportion of cancer survivors. Treatments used for other MN—intensive chemotherapy, hypomethylating agents, and the BCL‐2 inhibitor venetoclax—do not improve the survival of *TP53* ^mut^ MN patients meaningfully. Additionally, further development of many promising agents has been discontinued, highlighting the challenges. Widespread acknowledgment of these problems led to the recognition of *TP53* ^mut^ MN as a distinct entity in the 5th edition of the World Health Organization and International Consensus Classifications. However, critical discrepancies between the two classifications may lead to under‐ or overestimation of the prognostic risk. Here, we review recent advances in the biology, diagnosis, and treatment of *TP53* ^mut^ MN. The development of *TP53* ^mut^ MN is positioned at the intersection of age, hereditary predisposition, and anti‐cancer therapies. Precursor *TP53* ^mut^ clones can be detected years prior to the eventual leukemic transformation—raising the possibility of early intervention. We discuss the two classification systems and the bearing of the discrepancies between the two on timely and effective management. We provide novel evidence in the areas of discrepancies. Finally, we review the current therapeutic landscape and the obvious limitations of the currently used therapies.

## 1 Introduction

Alterations in tumor suppressor gene *TP53—*the “guardian of the genome”—are the most prevalent abnormalities in human cancers and are generally associated with poor outcomes [1]. *TP53* alterations are relatively uncommon in myeloid neoplasms (MN), accounting for 8%–10% of myelodysplastic syndrome (MDS) and 10%–12% of acute myeloid leukemia (AML) cases [2, 3, 4, 5, 6]. Despite that, it remains a vexing challenge for three principal reasons: first, it is a highly aggressive leukemia with median survival of less than 1 year. Second, rapid advances in other types of MN have failed to translate into improved survival for this subset [7]. Effective treatments of *TP53* ^mut^ MN remain elusive, primarily due to the inherent chemorefractory nature [8, 9, 10]. Commonly employed treatments including intensive chemotherapies or hypomethylating agents (HMA) confer little benefit. While selective targeting of BCL‐2 with the BH3‐mimetic venetoclax has been revolutionary for other types of AML, it does not translate in meaningful improvement in survival in this subset [11, 12]. Moreover, the development of multiple novel agents aimed at *TP53* ^mut^ MN have been discontinued due to lack of efficacy [13]. Finally, *TP53* ^mut^ are highly enriched in older patients as well as those with secondary and therapy‐related MN (t‐MN) [14]. Therefore, with aging population and the success of anti‐cancer therapies, the incidence of *TP53* ^mut^ MN is expected to rise. Collectively, improving outcomes of *TP53* ^mut^ MN remains one of the greatest unmet challenges.

Wide acknowledgement of these challenges led to the recognition of MN harboring *TP53* ^mut^ as a separate entity by the 5th edition of the World Health Organization (WHO‐5) as well as International Consensus Classifications (ICC). The stated goals for the distinct grouping include the wider recognition of extremely poor prognosis, encouraging research, and facilitating clinical trial design—ultimately stimulating drug discovery. However, wide adoption has been challenging given critical differences between the guidelines.

The objectives of this review are to summarize advances in biology, diagnostic criteria, classification, and treatment options for *TP53* ^mut^ MN. We discuss the known precursor lesions of *TP53* ^mut^ MN—namely clonal hematopoiesis (CH) and clonal cytopenia of undetermined significance (CCUS)—and factors associated with leukemic transformation, though admittedly, the knowledge is rapidly evolving. Our special emphasis is on the challenges brought on by discrepancies in the diagnostic classifications and on reviewing our approach to diagnosis and management. Finally, we review the current therapeutic landscape and its obvious limitations.

### 1.1 Biology of TP53

The tumor protein p53, encoded by the *TP53* gene located on chromosome 17p13, is a 53‐kilodalton protein that was initially identified in virally transformed cells and was categorized as an oncogene. However, subsequent research established that wild‐type p53 functions as a tumor suppressor, inhibiting growth and oncogenic transformation [15].

*TP53* belongs to an evolutionarily highly conserved family of transcription factors: *TP53, TP63*, and *TP73*. The wild‐type p53 protein consists of 393 amino acids and contains several functional domains: two N‐terminal transactivation domains, a conserved proline‐rich domain, a central DNA‐binding domain (DBD), and a C‐terminus encoding its nuclear localization signals and an oligomerization domain required for the transcriptional activity.

#### 1.1.1 p53: A Master Regulator of Diverse Cellular Processes

**FIGURE 1:**
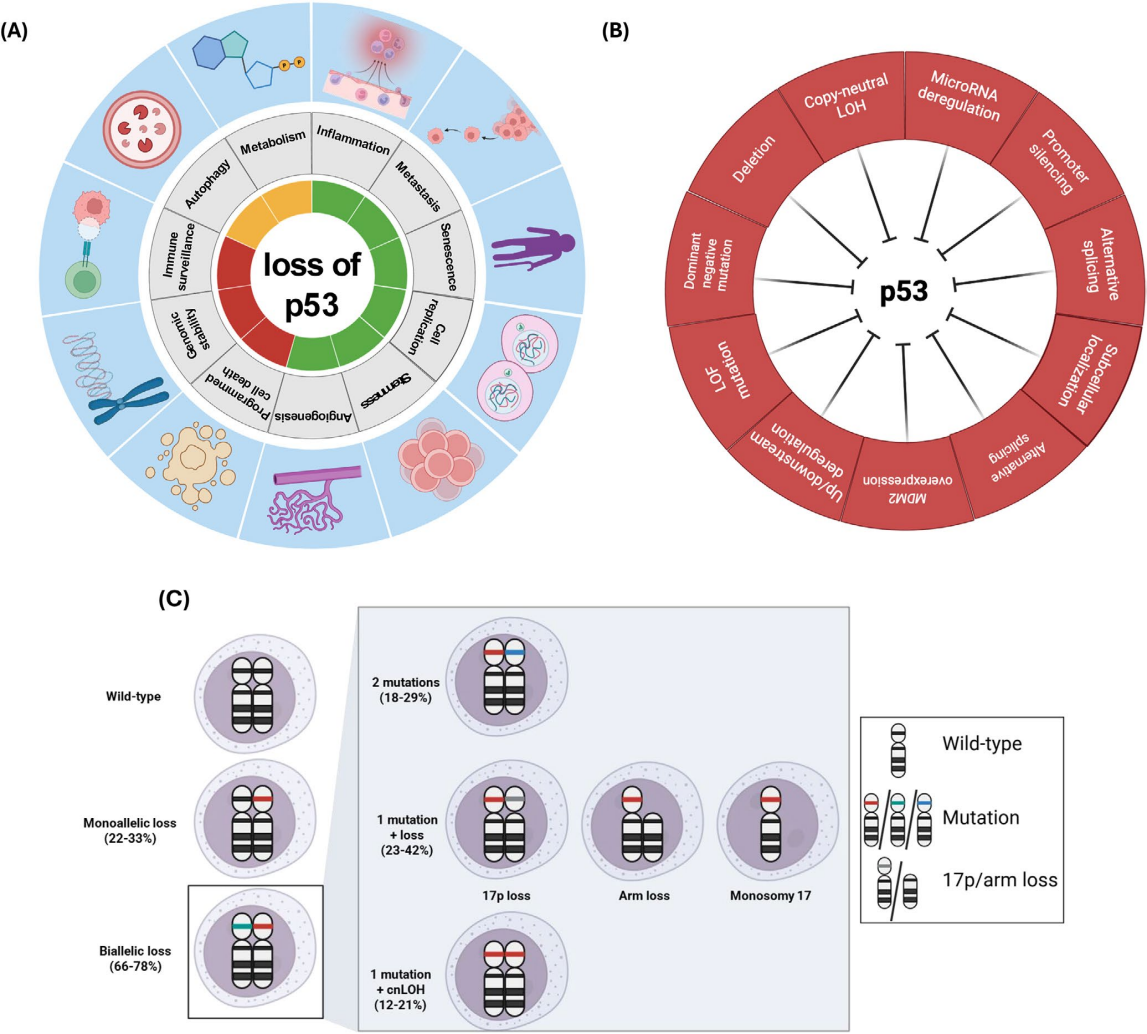
Biology of TP53 loss in cancers. (A) Consequences of the loss of TP53 in carcinogenesis. In the inner circle, red denotes suppressive effect, orange denotes mixed effect, and green denotes promoting effects; (B) Mechanisms of *TP53* inactivation; and (C) Common mechanisms of mono‐ and biallelic *TP53* inactivation in myeloid neoplasms (see text for the studies used to determine prevalence).

p53 has been implicated in a wide array of biological processes, including cell cycle arrest, senescence, apoptosis, autophagy, metabolism, and aging. It is also critical for maintaining genomic stability by balancing cell growth and cell arrest during genomic stress. p53 is usually present at a very low level in cells, and its short half‐life is regulated by post‐translational ubiquitination, acetylation, and phosphorylation. In a non‐stressed condition, p53 is ubiquitinated by the ubiquitin E3 ligase mouse double minute‐2 homolog (*MDM2*) leading to its proteasome‐mediated degradation [1]. A wide range of cellular stressors, including oncogenic, hypoxic, or DNA damage, inhibits *MDM2*, inhibiting ubiquitination of p53 and stabilizing it in tetrameric form. The tetrameric p53 induces the transcription of a diverse set of genes, turning on a highly complex anti‐proliferative transcriptional program that regulates virtually all known cellular functions (Figure 1A) [1, 16, 17, 18, 19]. This central location of the “*TP53*‐*MDM2* axis”—in part—explains why *TP53* alterations are the most common abnormalities in cancers [1]. p53 also plays a crucial role in maintaining genomic integrity, and regulating quiescence, self‐renewal, and differentiation of hematopoietic stem cells (HSC)—protecting against leukemogenesis [20, 21].

*TP53* ^mut^ affects p53's tetrameric conformations, impairing its ability to bind to transcriptional targets [22]. Hence, missense mutations along with truncating mutations and/or loss‐of‐heterozygosity/copy neutral loss‐of‐heterozygosity (LOH/cnLOH) can lead to loss of function, causing an inability to trigger p21, downregulation of genes associated with apoptosis, and upregulation of proteins involved in cell‐cycle progression and those involved in DNA‐damage repair (DDR). Murine studies also reported gain‐of‐function of missense mutations by demonstrating neomorphic protein–protein interactions of mutant p53 with other transcription factors. Moreover, a relative competitive fitness advantage of HSCs carrying missense mutations in the DBD over HSCs with monoallelic *TP53* inactivation was observed [23]. Finally, missense *TP53* ^mut^ can also exert a dominant‐negative effect on the wild‐type protein by forming tetramers with wild‐type p53, thus disrupting its transcriptional activity [23].

#### 1.1.2 Mechanisms of *TP53* Inactivation

While *TP53* ^mut^ is the most common mechanism of inactivation, other mechanisms include the *TP53* deletion, alternative splicing, post‐translational modification, micro‐RNA mediated degradation, or *MDM2*/*MDM4* upregulation, *ARF* downregulation (Figure 1B). Two‐thirds of *TP53* ^mut^ lead to biallelic inactivation either due to biallelic mutations or a single mutation with LOH/cnLOH of the wild‐type allele (Figure 1C). In MN, the mechanisms of biallelic *TP53* inactivation include: ≥ 2 mutations in 18.4%–28.8% (median 22.6%), 1 mutation with deletion of the trans allele in 22.5%–42.2% (median 33%), and 1 mutation with concomitant cnLOH in 12.2%–20.6% (median 17%) of patients [8, 24, 25, 26]. (and Shah et al., under review).

## 2 Pathogenesis of *TP53*‐Mutated Myeloid Neoplasms

Relative infrequency of t‐MN and long latency from cytotoxic therapies suggest the role of factors beyond CH in leukemic transformation. The incidence of t‐MN is 0.5%–8% following cytotoxic exposures for various cancers. Second, median latency from the diagnosis of primary cancer to t‐MN diagnosis is approximately 7 years, with 54% and 29% diagnosed ≥ 5 and ≥ 10 years from primary malignancy, respectively [27].

Recent advances in sequencing technology have revolutionized our understanding of the occurrence of clones in myeloid driver genes with preserved blood counts (CH) or clonal CCUS. The explosion in our understanding of these precursor lesions has provided critical insight into leukemogenesis, including the origin and leukemic transformation of *TP53* ^mut^ clones.

That *TP53* ^mut^ are early leukemogenic mutations is now well established. *TP53* ^mut^ constitutes 4%–5% of all CH [28, 29, 30, 31] and paired deep‐sequencing at the time of *TP53* ^mut^ t‐MN and at/before primary malignancy demonstrated identical *TP53* ^mut^ clone in t‐MN in a subset of cases. A seminal study demonstrated that mutational burden in the genomic region containing *TP53* was comparable between t‐AML and de novo AML, suggesting that it was unlikely that chemotherapy directly induced the *TP53* ^mut^ [32]. Indeed, in a smaller subset, the eventual *TP53* ^mut^ clone was detected even prior to the institution of any cytotoxic therapies. Combined, these observations strongly suggest preexistence of the clone that gained a fitness advantage under the selective pressure [32].

Later, larger studies confirmed these observations in the context of diverse primary malignancies and cytotoxic exposures [33, 34, 35, 36]. For example, in a case–control study of cancer patients treated with cytotoxic therapy, 36% of cases of t‐MN developed harbored *TP53* ^mut^. Paired sequencing of PB/BM samples at the time of primary cancer diagnosis showed somatic *TP53* ^mut^ at VAF 0.92%–22.3% in all evaluable cases [36]. Similarly, lymphoma patients undergoing autoHCT who later developed t‐MN, all 4 *TP53* ^mut^ MN cases had pre‐existing *TP53* ^mut^ clones at a low (0.9%–12%) VAF prior to autoHCT [33].

A study of 5978 patients with nonhematological cancers showed a 2.8‐fold higher risk of *TP53* ^mut^ CH in patients who received cytotoxic therapies. The risk differed based on the type of therapies: platinum (2.1‐fold), radiation therapy (1.8‐fold) and taxane (1.9‐fold). Another study confirmed some of these findings: chemotherapy—particularly platinum and topoisomerase II inhibitors—preferentially selected for mutations in DDR genes, including *TP53* [33, 35]. Radiation therapy was associated with the selective growth of *TP53* ^mut^ and in those developing *TP53* ^mut^ t‐MN, the *TP53* ^mut^ clone was the dominant clone, suggesting clonal selection of *TP53* ^mut^ under the strong cytotoxic pressure [35].

Thus, while it is clear that at least a subset of *TP53* ^mut^ MN is an end‐result clonal expansion/evolution of the preexistent *TP53* ^mut^ clone. However, the presence of *TP53* ^mut^ is necessary and sufficient for the development of *TP53* ^mut^ MN is not well understood. For example, in contrast to the cancer studies presented above, a population‐based study of 438 890 participants showed that *TP53* ^mut^ CH/CCUS did not increase the risk of subsequent MN (HR 0.94, *p* = 0.87). In murine studies, *TP53* ^mut^ HSC can promote self‐renewal but failed to induce overt transformation into leukemia, indicating that the mere presence of the *TP53* ^mut^ clone may be insufficient to initiate leukemia [20, 37] and additional selection pressure may be necessary for clonal expansion/evolution and transformation. Therefore, HSC‐intrinsic factors discussed above, including the cooperation of *BRCA1*/*2* PGV with *TP53* ^mut^ and the haploinsufficiency of genes mapped on the minimally deleted region on chromosome 5q [35]. In addition, a single‐cell multi‐omics study aptly demonstrated that a chronic inflammatory microenvironment confers a fitness advantage and promotes evolution of *TP53* ^mut^ while suppressing *TP53* ^wt^ HSC [38].

Collectively, emerging evidence suggests a diverse risk of leukemic transformation of *TP53* ^mut^ clones and that cooperation of HSC‐intrinsic factors such as genetic predisposition with HSC‐extrinsic factors such as genotoxic therapies and the bone marrow microenvironment can potentially shape clonal evolution and expansion. Factors associated with leukemic transformation of precursor states—CH and CCUS—are an area of active research.

### 2.1 *TP53* Mutations Are Associated With Immunosuppressive Milieu

In addition to the immense impacts on the leukemic cells, *TP53* ^mut^ modulates diverse aspects of the innate and adoptive immune system—further contributing to leukemogenesis. An elegant study showed that *TP53* ^mut^ clones reshape the microenvironment conducive to their survival, chemoresistance, and immune evasion. *TP53* ^mut^ MN HSCs have a higher expression of programmed death ligand‐1 (PD‐L1), which in turn is associated with c‐Myc upregulation and miR‐34a downregulation. Furthermore, the bone marrow of *TP53* ^mut^ MN harbors a reduced number of cytotoxic and helper T‐cells and natural killer (NK) cells, but shows expansion of highly immunosuppressive regulatory T‐cells (T_regs_) and myeloid‐derived suppressor cells—all suggestive of a highly immunosuppressed BM milieu [39]. *TP53* ^mut^ AML were associated with increased numbers of activated B‐cells, effector memory CD4^+^ T‐cells, central memory CD8^+^ T‐cells, and two NK‐cell‐rich clusters [40].

Collectively, these results suggest a profound immune dysregulation, with features of immune senescence and an overall immune evasive phenotype which could be potentially leveraged to develop immunotherapy for *TP53* ^mut^ MN. However, modest responses to immune checkpoint inhibitors (ICI) [41, 42, 43], and recent failures of anti‐CD47 monoclonal antibody [44], as well as monoclonal antibody against T‐cell Immunoglobulin Mucin (TIM)‐3 (sabatolimab) [45] suggest that overcoming the immunosuppressive *milieu* is a critical impediment to developing successful therapies (Table 1).

**TABLE 1:** Clinical and laboratory studies of *TP53*‐mutated (*TP53* ^mut^) myeloid neoplasms (MN).

Characteristics	Study					
	Bahaj [25]	Grob^a^ [8]	Haase^b^ [10]	Kaur^c^ [46]	Shah^d^	Weinberg^e^ [47]
*N*	1010	230	186	173	580	247
Age in years, median	71	62	70	67.9	68.6	70
Male (*n*, %)	536 (53%)	136 (59%)	108 (58%)	104 (60%)	366 (63%)	129 (52%)
BM Blasts < 5% (*n*, %)	NR	0 (0%)	54 (29%)	0 (0%)	194 (33.4%)	43 (17.4%)
BM blasts 5%–9% (*n*, %)	NR	44 (19.1%)	59 (31.7%)	0 (0%)	75 (12.9%)	37 (15.0%)
BM blasts 10%–19% (*n*, %)	NR		65 (34.9%)	36 (20.8%)	92 (15.9%)	54 (21.9%)
BM blasts ≥ 20% (*n*, %)	NR	186 (80.9%)	3 (1.6%)	137 (79.2%)	219 (31.8%)	113 (45.7%)
Prior therapy (%)	NR	NR	NR	NR	278 (47.9%)	106 (42.9%)
Hemoglobin (g/dL), median	9.1	NR	9.2	7.9	8.9	8.5
WBC (10^9^/L), median	5.1	NR	NR	NR	3	3
Platelets (10^9^/L), median	65	NR	47	40	55	50
Peripheral blasts %	NR	NR	NR	—	1	1

Abbreviations: ANC—absolute neutrophil count; BM—bone marrow; WBC—white blood cells.

^a^ MDS‐EB/AML cases only.

^b^ MDS with CK only.

^c^ Multihit *TP53* ^mut^ cases with blasts ≥ 10% only.

^d^ In revision.

^e^ MN with CK only.

## 3 Epidemiology of *TP53*‐Mutated Myeloid Neoplasms

The Pan‐Cancer cohort confirmed *TP53* as the most commonly altered gene across various cancers. The prevalence of its alterations varies widely—from ~95% in serous ovarian cancers to ~2% in renal cancers [4]. MN are positioned at the lower end of this spectrum, with 7.5% of MDS and AML harboring *TP53* ^mut^ [3, 4]. However, *TP53* ^mut^ is enriched in 3 scenarios in MN: (1) older individuals; (2) individuals with germline pathogenic variants (PGV) including Li‐Fraumeni syndrome, *BRCA1*, and *BRCA2* carriers; and (3) patients who had prior cytotoxic or immunosuppressive therapies.

1. Older age. The median ages of *TP53* ^mut^ AML and MDS are 67 and 73 years, respectively. *TP53* ^mut^ AML is significantly more common in patients ≥ 60 compared to < 60 years of age (7% vs. 2%) and within the ≥ 60 cohort, the frequency of *TP53* ^mut^ AML increases with age: 16%, 19%, 17%, and 50% in 61–70, 71–80, 81–90, and 91–100 years, respectively. On the other hand, no such difference was seen for MDS [14].
2. Pathogenic germline variant carriers. *TP53*‐deficient tumors may be enriched in patients with PGV—especially those in the DDR genes. It is thought that TP53 dysfunction is integral to *BRCA1/2‐*associated tumorigenesis, which could explain the enrichment of *TP53* ^mut^ t‐MN in *BRCA1/2* PGV carriers. The enrichment is likely heightened by the use of cytotoxic therapies for treating primary cancer in carriers of DDR genes [48]. *TP53* ^mut^ t‐MN are indeed more prevalent in *BRCA1/2* PGV carriers of breast and non‐breast/ovarian cancer survivors [49, 50]. Ovarian cancer patients with *BRCA1/2* PGV are 6.2‐fold more likely to harbor *TP53* ^mut^ CH compared to *BRCA1/2* wild‐type individuals [51]. At least a subset of pre‐existing *TP53* ^mut^ clones expanded during poly (ADP‐ribose) polymerase inhibitors (PARPi) therapy, suggesting a potential mechanistic link [35]. A study of 53 t‐MN patients that included 12 patients with prior breast or ovarian cancers found that approximately half harbored PGV in DDR genes [52].

Along the same lines, somatic inactivation of the trans allele—via the LOH, cnLOH, or copy gain LOH (cgLOH)—is a common occurrence in patients with Li–Fraumeni syndrome (LFS)—rare autosomal dominant cancer predisposition syndrome caused by PGV in *TP53*. LFS patients are at an increased risk of developing multiple cancers, including ~4% incidence of hematological malignancies [53]. In tumors arising in LFS individuals, LOH/cnLOH of *TP53* was seen in a majority (86%) of cases that appeared to have occurred as early as in utero [54].

3 Prior cytotoxic and immunosuppressive therapies. Cancer survivors are at a 4.7‐fold higher risk of developing leukemia compared to the general population. As the effectiveness of cancer‐directed therapies has improved survival, long‐term complications of these therapies have come to focus. For instance, the incidence of t‐MN has risen over the last two decades: from 0.04/10 000 new cases between 2001 and 2007 to 0.20/100 000 new cases between 2008 and 2014 [55].

**FIGURE 2:**
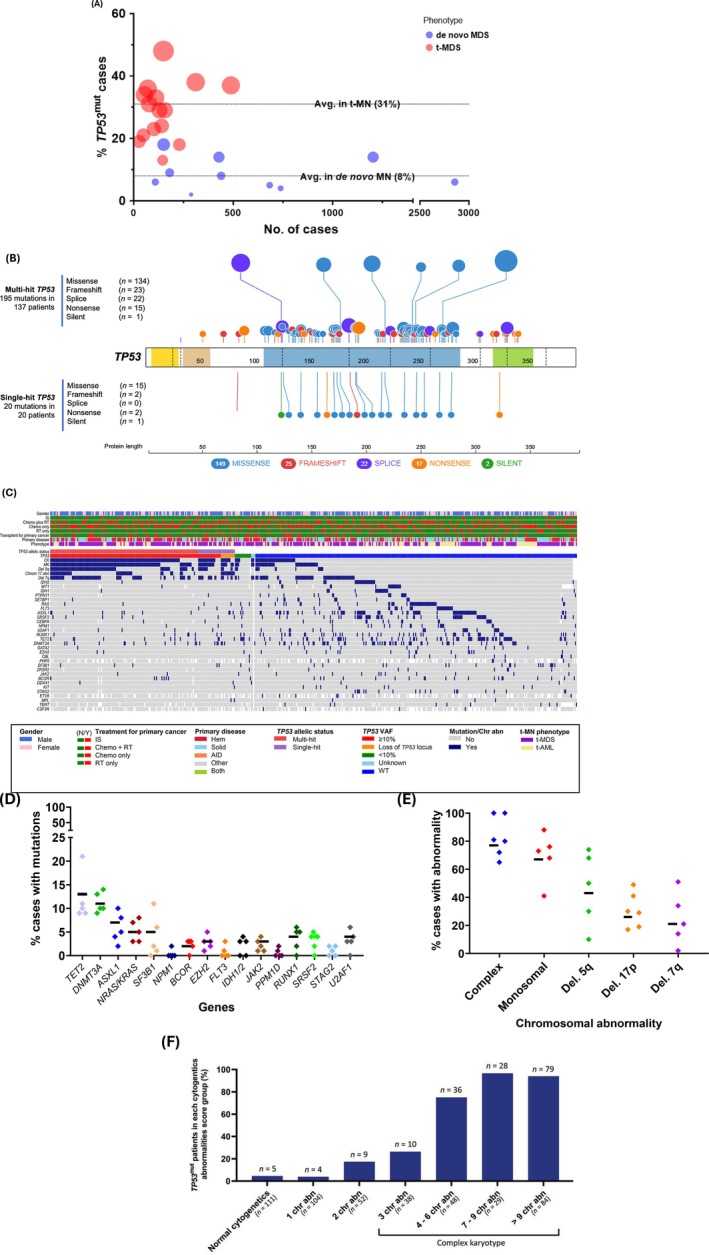
Molecular and chromosomal abnormalities in *TP53*‐mutated (*TP53* ^mut^) myeloid neoplasms (MN). (A) *TP53* ^mut^ is enriched in therapy‐related compared to de novo MN; (B) Lollipop Plot of *TP53* ^mut^ in therapy‐related myeloid MN (t‐MN, 253 variants). Missense mutations are predominant in *TP53* ^mut^ MN, with 85% of cases harboring one or more missense mutations. As with other cancers, > 80% of *TP53* ^mut^ in MN localize to the DNA‐binding domain (DBD, 100–300 amino acids) and localize to 6 hotspot sites (R175, H179, Y220, G245, R248, R273); (C) OncoPlot for *TP53* ^mut^ t‐MN (*N* = 182 patients). *TP53* ^mut^ MN are associated with a paucity of co‐mutations in other myeloid driver genes and a very high prevalence of chromosomal abnormalities; (D) Percentage cases with co‐mutations in the most commonly co‐mutated genes; (E) Percentage cases with chromosomal abnormalities; and (F) In myeloid neoplasms, *TP53* ^mut^ is enriched in complex karyotype. AID—autoimmune disease; chemo—chemotherapy; chr. abn.—chromosomal abnormality; del.—deletion; Hem—hematological malignancy; IS—immunosuppression; RT—radiotherapy; solid—solid tumor; t‐AML—therapy‐related acute myeloid leukemia; t‐MDS—therapy‐related myelodysplastic syndrome; VAF—variance allele frequency; WT—wild‐type. Panel (A) source data from Singhal et al. [56] Panels (B), (C), and (F) are adapted from Shah et al. [57] In panels (D) and (E), each diamond represents a study, whereas the line (—) represents the weighted average across the studies when available.

*TP53* ^mut^ is enriched in t‐MN (13%–48%) compared to de novo MN (2%–18%) (average 31% vs. 8%, Figure 2A, Table 1) [2, 3, 4, 6, 8, 9]. Solid and hematological cancers precede t‐MN in approximately 40% and 50%, respectively; the remaining develop following treatment for autoimmune rheumatological diseases (AIRD) [58] or solid organ transplant. t‐MN developing in MM and ovarian cancer patients is significantly enriched with *TP53* ^mut^ [59]. Among treatments, autologous hematopoietic stem cell transplant (autoHCT) and chemotherapy exposure were associated with 2.2‐ and 2‐fold higher risk of *TP53* ^mut^ t‐MN compared to radiation exposure alone [27].

**TABLE 2:** Summary of the diagnostic criteria for myeloid neoplasms harboring *TP53* mutations (*TP53* ^mut^) between the 5th Edition of the World Health Organization (WHO‐5) and International Consensus Classifications (ICC).

Blast % category	WHO‐5	ICC	Comments
0%–9%	MDS with biallelic *TP53* inactivation: ≥ 2 *TP53* ^mut^ or 1 *TP53* ^mut^ with *TP53* copy number loss or cnLOH*	Multi‐hit: ≥ 2 *TP53* ^mut^ (each with VAF ≥ 10%) or 1 *TP53* ^mut^ (with VAF ≥ 10%) and (1) deletion involving the *TP53* locus at 17p**; (2) VAF ≥ 50%; or (3) copy neutral LOH at the 17p. Multi‐hit equivalent: One *TP53* mutation (VAF ≥ 10%) and a complex karyotype	Emphasis on “multi‐hit status” in both ICC and WHO‐5. ICC requires VAF ≥ 10%, WHO‐5 does not. In the context of 1 *TP53* ^mut^ (VAF ≥ 10%) and the absence of LOH information, CK is considered “multihit equivalent” by ICC but not by WHO‐5
10%–19%		MDS/AML with mutated *TP53*: *TP53* ^mut^ VAF ≥ 10%, regardless of allelic status	Continued emphasis on biallelic loss in WHO‐5, but not ICC. Continued emphasis on VAF ≥ 10% in ICC, but not WHO‐5.
≥ 20%	No distinct designation and included with other AML	AML with mutated *TP53*: *TP53* ^mut^ VAF ≥ 10%, regardless of allelic status	No separate designation in WHO‐5. Continued emphasis on VAF ≥ 10% in ICC.

Abbreviations: AML—acute myeloid leukemia; BM—bone marrow; cnLOH—copy neutral LOH, LOH—loss‐of‐heterozygosity; MDS—myelodysplastic syndrome; VAF—variant allele frequency.

* Per WHO‐5, copy number variation (CNV) analysis is required to confirm the loss of the *TP53* locus, as the mere detection of the 17p13.1 deletion is not sufficient.

** Per ICC, a cytogenetic deletion involving the *TP53* locus at 17p13.1 is adequate and does not mandate confirmatory CNV analysis.

In summary, emerging data suggest the propensity to develop *TP53* ^mut^ clones due to aging or hereditary predisposition, a transformation that is accelerated by cooperating genetic defects or iatrogenic selection pressure.

### 3.1 Emerging Therapies Associated With an Increased Risk of *TP53* ^mut^ MN

With increased utilization of targeted and immunotherapies, it was hoped that the incidence of t‐MN—and *TP53* ^mut^ MN—would decrease. Emerging data, however, suggest that many novel therapies are associated with an increased risk of both. The following section examines the evolving connection between non‐traditional anticancer therapies such as peptide receptor radionuclide therapies (PRRT), PARPi, and chimeric antigen receptor (CAR) T‐cell therapies with *TP53* ^mut^ MN and the commonalities.

Studying these connections is critical for two reasons: utilization of these therapies is expected to grow exponentially both in the breadth of indications as well as in volume. Second, t‐MN developing after the novel therapies shares some features including the proportion of *TP53* ^mut^ MN, chromosomal abnormalities, and outcomes comparable to the traditional cytotoxic therapies, whereas latency appears to be substantially shorter—deserving additional studies.

Peptide receptor radionuclide therapies (PRRT) such as Lutetium‐177‐Dotatate (^177^Lu‐Dotatate) and Lu 177 vipivotide tetraxetan (^177^Lu‐PSMA‐617) are targeted systemic radiopharmaceutical therapies that use beta particle‐emitting radionuclides linked to peptide analogs. ^177^Lu‐Dotatate is approved by the US Food and Drug Administration (FDA) for the treatment of somatostatin gastroenteropancreatic neuroendocrine tumors (GEP‐NETs). ^177^Lu‐PSMA‐617 is approved for the treatment of metastatic castration‐resistant prostate cancer (mCRPC). Approximately 1.4%–4.8% of patients treated with ^177^Lu‐Dotatate developed t‐MN, following a median latency of 3–4 years from the treatment [60, 61]. In a recent study describing 14 t‐MN following PRRT therapies, 2 multi‐hit *TP53* ^mut^ MDS developed at 17 and 18 months following the therapy. In addition, two patients had mutations in protein phosphatase magnesium‐dependent (*PPM*)‐1D gene, which were mutually exclusive [62].

A recent single‐center case series described 5 (1.3%) t‐MN developments among 381 (1.3%) patients treated with ^177^Lu‐PSMA, of which 3 had multi‐hit *TP53* inactivation and high‐risk karyotype in all cases [63].

PARPi are the standard‐of‐care therapy for ovarian, breast, or metastatic prostate cancer with known or suspected defective DDR mechanisms. PARP is involved in the repair of single‐strand DNA breaks, while DDR genes such as *BRCA1 and BRCA2* facilitate double‐strand DNA breaks. In a study of 298 *BRCA1/2* mutated cancer patients treated with olaparib, 6 (2%) t‐MN were noted [64]. A meta‐analysis of 28 randomized controlled trials comparing PARPi‐ (*n* = 5693) and placebo‐treated (*n* = 3406) patients showed that PARPi was associated with a 2.6‐fold higher risk of t‐MN (0.73% vs. 0.47%). Notable features included a short duration of PARPi exposure (median 9.8 months) and a strikingly short latency to develop t‐MN (median 20.3 months) following PARPi exposure [65]. Between 50%–75% of t‐MN following PARPi had *TP53* ^mut^, the majority being multi‐hit or multi‐hit equivalent [66, 67]. Enrichment of *TP53* ^mut^ t‐MN could partially be ascribed to a higher burden of *TP53* ^mut^ CH (64% vs. 14.3%) in PARPi‐treated patients compared to those who did not receive PARPi as well as clonal expansion during PARPi therapy [67]. Taken with the knowledge that a proportion of these patients carry cancer susceptibility PGV (discussed above), this may explain the high incidence of *TP53* ^mut^ t‐MN in this group.

Chimeric antigen receptor (CAR) T‐cell therapies have revolutionized the management of relapsed or refractory B‐cell acute lymphoblastic leukemia, CD19+ LPD, and MM. With wider utilization and longer follow‐up, an increased risk of secondary cancers—including t‐MN—is emerging and has been an area of immense focus recently [68, 69, 70, 71, 72, 73, 74, 75].

The cumulative incidence of t‐MN following CAR‐T therapy (*n* = 312) was 4%, 6%, and 9% at 1‐, 2‐, and 3‐years, respectively. Notably, 44.4% of t‐MN cases harbor *TP53* ^mut^. A striking feature of post‐CART t‐MN is the short latency (median 9.1 months from CART therapy) with 60% of t‐MN following CAR‐T [69] diagnosed within 1 year. This is in stark contrast to the much longer latency of 5–6 years following conventional cytotoxic therapies including autoHCT [33, 48, 76]. The short latency and high incidence of *TP53* ^mut^ following CAR‐T therapy were comparable between LPD and MM cases treated with CD19‐ and BCMA‐directed constructs—suggesting a potential mechanistic link between CAR‐T therapy and the subsequent MN. A higher proportion of *TP53* ^mut^ t‐MN can partly be explained by the high prevalence (37%–64%) of CH at baseline, predominantly involving *TP53* and *PPM1D* [70, 72]. The same *TP53* ^mut^ clone was detected in a subset of post CAR‐T t‐MN, suggesting that CAR‐T therapy may lead to rapid expansion of pre‐existing clones via hitherto unexplained mechanisms [70].

In summary, the study of t‐MN developing after novel therapies is an area of immense interest. Whereas comparable genetic and genomic features (including the proportion of multi‐hit *TP53* ^mut^) strongly suggest shared pathogenesis, these novel modalities are used in conjunction with or following cytotoxic therapies. Therefore, whether the above therapies induce mutations or cause the expansion of the pre‐existing mutation is unclear. Further research is needed to isolate the impact of these therapies on *TP53* ^mut^ MN development and the mechanisms thereof.

## 4 Clinical and Molecular Characteristics of *TP53*‐Mutated Myeloid Neoplasms

### 4.1 The Landscape of *TP53* Mutations in MN

Deletion of the entire chromosome 17 or 17p13.1 across the *TP53* locus and truncating mutations can lead to *TP53* loss of function. However, unlike for most other suppressor genes, missense mutations are predominant in *TP53* ^mut^ MN, with 77% being missense, 9% frameshift, 6% splice, 6% nonsense, 2% deletion, and 1% silent variants (Figure 2B). As with other cancers, > 80% of *TP53* ^mut^ in MN localize to the DNA‐binding domain (DBD, 100–300 amino acids) and localize to hotspot sites [77]. MN, including t‐MN, harbor a distinct profile of mutational hotspots when compared to other cancers. An analysis of the International Agency for Research on Cancer TP53 database showed that 6 commonest mutations (R175, G245, R248, R249, R273, and R282) account for 27.7% of all *TP53* ^mut^ across cancers [77]. In contrast, the mutational spectrum of all MN and t‐MN showed a consistently distinct pattern, with R175, Y220, M237, R248, R273, and R282 being the most common variants [25, 57]. The biological basis and impact of the mutational characteristics (e.g., location, hot spot vs. not) in MN remain poorly understood.

### 4.2 The Pattern of Co‐Mutations in *TP53*‐Mutated MN

Given the well‐established role of *TP53* in DDR, it may be assumed that *TP53* ^mut^ MN would have a higher mutation burden. However, a striking feature of *TP53* ^mut^ MN compared to *TP53* ^wt^ MN is the paucity of co‐mutations in myeloid driver genes [57, 78]. More than half (52%) of *TP53* ^mut^ t‐MN harbor no known co‐mutations compared to 17.3% of *TP53* ^wt^ t‐MN [9] with an average number of co‐mutations being lower in biallelic *TP53* ^mut^ compared to *TP53* ^wt^ MN [25] (0.8 vs. 2.1 per case, Figure 2C,D). In *TP53* ^mut^ t‐MN, ≥ 2 co‐mutations were noted in only 20.3% of *TP53* ^mut^ cases compared to 63.7% of *TP53* ^wt^ t‐MN [9]. The paucity of co‐mutations is across the genes/pathways including signaling pathway (*KIT*, *FLT3*, *WT1*), epigenetic modifiers (*DNMT3A*, *TET2*, or *ASXL1*), or spliceosome (*U2AF1*, *SF3B1*, *SRSF2*, or *ZRSR2*). On the other hand, alterations in *ETV6* and *NF1* are enriched in *TP53* ^mut^ MN [78]. Even among the MN harboring *TP53* ^mut^, “*TP53*‐driven” tumors (i.e., multi‐hit *TP53* ^mut^) were characterized by a paucity of co‐mutations compared to single‐hit *TP53* ^mut^ [24, 57], suggesting that multi‐hit TP53 inactivation is a biologically distinct entity compared to both single‐hit *TP53* ^mut^ and *TP53* ^wt^ MN.

In the absence of a clear co‐mutational pattern associated with survival in *TP53* ^mut^ MN, scoring systems such as Evolutionary action for *TP53* (EAp53) [79, 80, 81] and EPI‐6 scores [46] have been proposed. EAp53 is a computational approach initially developed for head and neck squamous cancers that has been applied to *TP53* ^mut^ MN with variable success [80, 81]. Kaur et al. recently proposed the EPI‐6 score that accounts for alterations in one or more of the 6 genes (*CUX1*, *U2AF1*, *EZH2*, *TET2*, *CBL*, or *KRAS*) that predicted inferior 2‐year survival in venetoclax and HMA‐treated patients [46]. The lack of independent validation has limited wider adoption of these scoring systems in routine practice.

### 4.3 Chromosomal Abnormalities in *TP53*‐Mutated MN

MN are remarkably “silent” genomically compared to other cancers [4, 82], and chromosomal abnormalities are noted in only 40%–50% of MN [3, 25, 83, 84]. Given the role of *TP53* as “the guardian of the genome,” it is conceivable its loss is associated with genomic alterations. In the absence of functional p53, cells are unable to effectively repair DNA‐damage—culminating into large chromosomal alterations. Indeed, a striking feature of *TP53* ^mut^ MN is the pervasiveness of cytogenetic abnormalities including highly complex genomic aberrations and chromothripsis [24, 57].

Metaphase cytogenetic studies demonstrate the enrichment of complex karyotypic (CK; defined as ≥ 3 chromosomal abnormalities in the absence of AML‐defining translocations and/or inversions) abnormalities in 70%–80% of *TP53* ^mut^ MN compared to 10% of *TP53* ^wt^ MN (Figure 2E) [25]. Similarly, chromosomal abnormalities are more prevalent in *TP53* ^mut^ t‐MN compared to *TP53* ^wt^ t‐MN—50%–60% of *TP53* ^wt^ t‐MN harbor a normal karyotype, whereas only 12.4% of *TP53* ^mut^ t‐MN have a normal karyotype. Recurrent cytogenetic abnormalities, including deletion 5q, deletion 7q, and loss of 17p, are all common in *TP53* ^mut^ MN. Conversely, the frequency of *TP53* ^mut^ increases from 4.5% in normal karyotype cases to 17.3% in the presence of two chromosomal aberrancies and 76.8% in the presence of CK. Even within the CK group, *TP53* ^mut^ is further enriched with the increasing number of cytogenetic abnormalities: from 26.3% in cases with 3 chromosomal abnormalities to 75%, 96.6%, and 94% in cases with 4–6, 7–9, and > 9 chromosomal aberrancies (Figure 2F).

Finally, the association of CK on survival in *TP53* ^mut^ is context dependent: in a cohort of de novo *TP53* ^mut^ MN, the presence of CK was associated with inferior survival compared to non‐CK *TP53* ^mut^ MN [8]. Conversely, in a cohort of 287 MN harboring CK, 83% harbored *TP53* ^mut^ and were an independent risk factor for inferior survival [47]. The impact of CK appears to be limited to MDS with no increased blasts but not in cases with increased blasts [26]. These observations do not translate to *TP53* ^mut^ t‐MN, wherein survival is poor regardless of CK (7.3 vs. 8.3 months) [57].

Finally, p53 suppresses chromosome shattering and rearrangement events known as chromothripsis. Chromothripsis is characterized by massive genomic rearrangements that are often generated in a single catastrophic event and an oscillating pattern of DNA copy‐number levels in one or a few chromosomes [82, 85]. Consequently, the loss of p53 facilitates accumulation and permits the survival of aneuploid cells. Pan‐Cancer Analysis of Whole Genomes (PCAWG) across all tumor types showed that *TP53* ^mut^ was associated with a 1.5‐fold higher risk of chromothripsis compared to *TP53* ^wt^ (38% vs. 24%) [82]. In a study of AML with CK, chromothripsis was noted in 35% of cases, and chromosomes 7 (10%), 3 (9%), and 12 (9%) were the most commonly involved targets. Chromothripsis is independently associated with a 2.5‐fold higher risk of death in AML [85].

### 4.4 Impact of *TP53* Mutations in MDS With Isolated Deletion of the Long Arm of Chromosome 5

MDS with isolated deletion of the long arm of chromosome 5 [MDS‐del(5q)] is a distinct sub‐entity of MDS characterized by superior outcomes, a favorable response to lenalidomide, and enrichment of *SF3B1* mutations [86]. *TP53* ^mut^ are enriched in MDS‐del(5q) cases (18%–20% vs. 5%–10% in other MDS) and are further enriched in the leukemic phase [87, 88, 89]. *TP53* ^mut^ are also enriched in therapy‐related compared to de novo MDS‐del(5q) (30% vs. 19%) [89], but a significantly lower proportion harbors multi‐hit inactivation compared to non‐del(5q) MDS (25% vs. 70%–75%) [16, 88].

*TP53* ^mut^ is associated with inferior survival and increased risk of AML progression in this otherwise favorable‐risk category and thus represents cases at a crossroad of the opposite ends of the prognostic spectrum [90]. It is thought that genes in the minimally deleted region on chromosome 5q, including casein kinase‐1 alpha 1 (*CSNK1A1*), Early Growth Response 1 (*EGR1*), and *APC*, cooperate with *TP53* ^mut^ to confer a survival advantage in HSC and progression to AML [91, 92]. Inhibition of CSNK1A1 induces p53‐mediated apoptosis; selective lenalidomide‐mediated degradation of *CSNK1A1* is more toxic to normal HSC, providing the competitive advantage seen for the *TP53* ^mut^ clone [59].

Recent studies identified multi‐hit *TP53* ^mut^ or monoallelic *TP53* ^mut^ with VAF ≥ 20 [88] or ≥ 22% [93] as the subset with poor survival. In contrast, monoallelic *TP53* ^mut^ cases with VAF < 22% had survival and AML progression risks comparable to *TP53* ^wt^ MDS‐del(5q) [88]. While highly effective, lenalidomide therapy can lead to selective expansion of *TP53* ^mut^ clones [59] raising the possibility of leukemic transformation.

In conclusion, integrating *TP53* ^mut^ allelic status and VAF threshold into diagnostic, monitoring, and management strategies can better stratify risk and guide treatment decisions in MDS‐del(5q).

### 4.5 The Impact of Multihit *TP53*‐Mutations in Myeloproliferative Neoplasms

In the current form of WHO‐5 and ICC, *TP53* ^mut^ MN does not include myeloproliferative neoplasms [86, 94]. A recent study evaluated the impact of *TP53* ^mut^ (VAF ≥ 2%) in myeloproliferative neoplasm (MPN) cases. Accelerated/blast (AP/BP) cases harboring *TP53* ^mut^ had a significantly shorter survival compared to *TP53* ^wt^ MPN‐BP/AP, regardless of the allelic status or VAF. On the other hand—reminiscent of the *TP53* ^mut^ MDS—multihit *TP53* ^mut^ had a shorter survival compared to non‐multihit *TP53* ^mut^ for chronic phase myelofibrosis [95].

## 5 Diagnosis of *TP53*‐Mutated Myeloid Neoplasms

*TP53* ^mut^ is associated with poor survival in all MN including MDS, AML, MDS/MPN, and MPN [6, 8, 9, 96, 97] (and Tefferi et al., in print). Recently proposed the 5th edition of the WHO classification (WHO‐5) and International Consensus Classification (ICC) acknowledged *TP53* ^mut^ MDS and AML as a distinct entity in recognition of the biological homogeneity, poor survival, and biological and clinical distinctness from *TP53* ^wt^ MN [86, 98]. Regardless of the blast percentage—*TP53* ^mut^ MN shares striking similarity in the enrichment of cytogenetic abnormalities, paucity of co‐conspiring mutations, and most importantly, outcomes [8, 9].

Classification of *TP53* ^mut^ MN can be seen as a two‐step process: First, the assignment of *TP53* hit status and second, the integration of the hit status with blast % category. *TP53* ^mut^ is usually established by targeted sequencing analysis covering at least exons 4–11. Molecular pathology methods capable of detecting LOH include fluorescence in situ hybridization (FISH), single nucleotide polymorphism (SNP) array, a specialized NGS panel designed to detect 17p LOH, and WGS. cnLOH can be detected by SNP array, the specialized NGS panel, or WGS. The presence of ≥ 2 mutations usually targets both alleles and is considered biallelic/multi‐hit events. Similarly, the presence of one *TP53* ^mut^ with VAF ≥ 50% is considered to be presumptive (not definitive) evidence of LOH/cnLOH (Table 2).

### 5.1 Review of the 5^th^ Edition of the WHO and International Consensus Classification for *TP53*‐Mutated Myeloid Neoplasms

**FIGURE 3:**
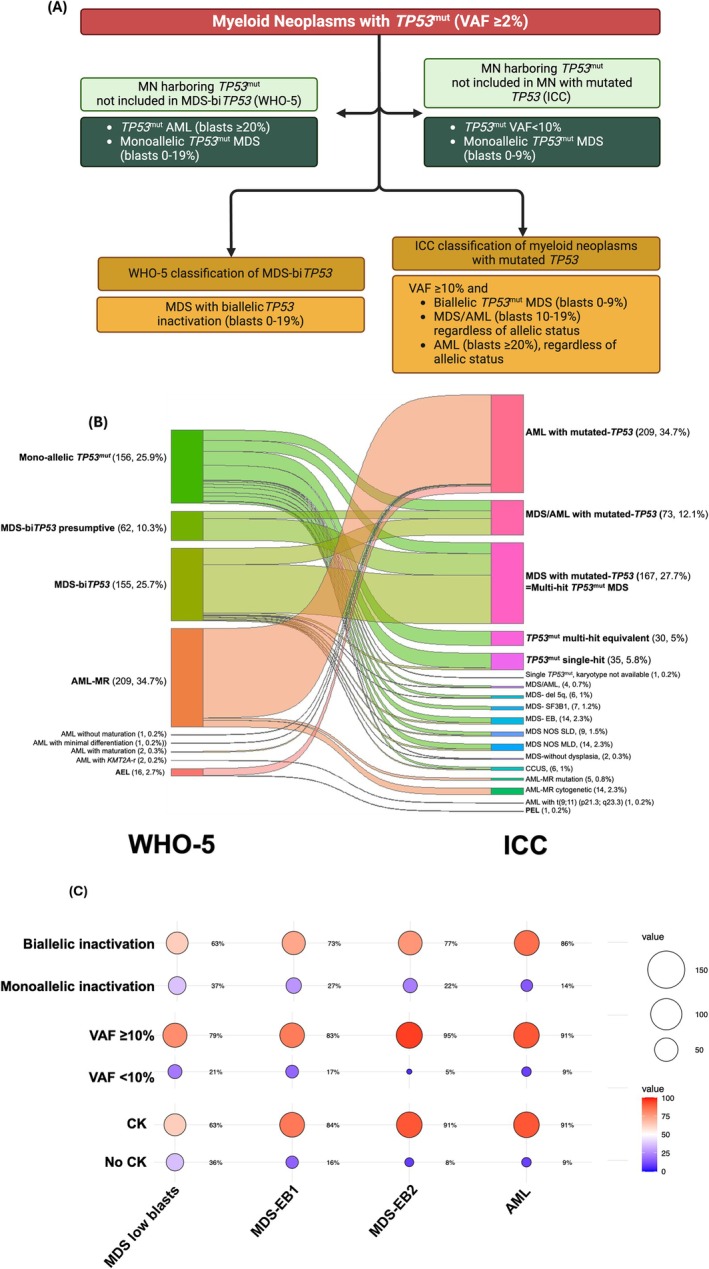
Myeloid neoplasms (MN) harboring *TP5*3 mutations (*TP53* ^mut^) are classified discrepantly using the 5th edition of the World Health Organization (WHO‐5) and International Consensus Classifications (ICC). (A) Schema representing the 5th edition of the WHO (WHO‐5, for MDS with bi*TP53*) and International Consensus Classifications (ICC, myeloid neoplasms with mutated *TP53*); (B) Retrospective application of MN harboring *TP53* ^mut^ (variant allele frequency ≥ 2%) classifies a 67.5% of the cohort discrepantly; (C) interaction between blasts, allelic status, complex karyotype, and VAF. AEL—acute erythroleukemia; AML—acute myeloid leukemia; AML‐MR—AML myelodysplasia related; CCUS—clonal cytopenia of undetermined significance; CK—complex karyotype; EB—excess blasts; EB—excess blasts; LB—low (< 5% blasts); MDS—myelodysplastic syndrome; MLD—multilineage dysplasia; MN—myeloid neoplasms; PEL—pure erythroid leukemia; SLD—single‐lineage dysplasia; VAF—variant allele frequency; VAF—variant allele frequency. Panel B is adapted from Shah et al. (under review).

The stated goals for distinct classification of *TP53* ^mut^ MN include the wider recognition of extremely poor prognosis, encouraging research, and facilitating clinical trial design—ultimately stimulating drug discovery. In practice, however, the adoption of the classifications has been challenging due to discrepant criteria. For example, in a study of 603 MN harboring *TP53* ^mut^ (VAF ≥ 2%), 64% and 20.4% of *TP53* ^mut^ MN cases would not be classified as *TP53* ^mut^ MDS or MN by WHO‐5 and ICC, respectively (Figure 3A). Moreover, 407 (67.5%) would be classified discrepantly (Figure 3B) [99]. Another study (*n* = 188) confirmed these findings to demonstrate that 64% of cases would be classified differently by WHO‐5 and ICC [100]. As both classifications are increasingly used and governing clinical practice, the differences between the two classifications can lead to either under‐ or overestimation of the prognostic risk and inconsistencies in treatment decisions.

The critical differences between the two classifications are driven by the assigned importance to morphological assessment, *TP53* ^mut^ AML, allelic status, and blast percentage in cases with MDS, complex karyotype, 17p13.1 deletion detected on metaphase cytogenetic, and *TP53* ^mut^ VAF threshold (Table 3). The WHO‐5 classification includes only biallelic *TP53* ^mut^ MDS, whereas the ICC includes MDS and AML in a distinct category of *TP53* ^mut^ MN.

**TABLE 3:** Areas of discrepancy between the 5th edition of the World Health Organization (WHO‐5) and International Consensus Classifications (ICC) for the classification of *TP53*‐mutated (*TP53* ^mut^) myeloid neoplasms (MN).

No.	Area of discrepancy	WHO‐5: MDS with biallelic *TP53* inactivation	ICC: Myeloid neoplasms with mutated *TP53*	Emerging evidence	Conclusions
1	Acute myeloid leukemia (AML)	No separate category, most cases would be classified as AML‐MR.	Separate category (BM/PB blasts ≥ 20%), includes PEL	Survival of *TP53* ^mut^ AML is significantly poor compared to *TP53* ^wt^ AML‐MR (4.7 vs. 18.3 mo.; *p* < 0.0001) [99, 100].	*TP53* ^mut^ AML should be included in *TP53* ^mut^ MN.
2	Variant allele frequency (VAF) threshold	None	VAF ≥ 10%	In cases with *TP53* ^mut^ VAF < 10%: Survival of AML and multi‐hit *TP53* ^mut^ “MDS” was significantly poor compared to their counterpart with single hit, and comparable to cases with VAF ≥ 10% (14.1 vs. 48.8 vs. 7.8 mo., *p* < 0.0001).	“Multihit” *TP53* ^mut^ with VAF 2% to < 10% should be included in *TP53* ^mut^ MN.
3	Interaction of blast % categories with allelic status	Requires demonstration of biallelic inactivation for blasts 0%–19%	Requires demonstration of multi‐hit status for blasts 0%–9%	Survival of monoallelic MDS ≥ 5% is comparable to biallelic inactivation [99] and (Shah et al., under review).	Monoallelic *TP53* ^mut^ MDS with blasts ≥ 5% should be included in *TP53* ^mut^ MN.
4	Complex karyotype (CK) as multihit equivalent	CK is not considered a multi‐hit equivalent	CK is considered multi‐hit equivalent	Survival of single *TP53* ^mut^ VAF < 50% with CK is comparable to 17p deletion on karyotype (10.4 vs. 11.0 mo.; *p* = 0.39) and poorer than monoallelic *TP53* ^mut^ without 17p loss or CK (33.4 mo.; *p* < 0.0001) [99].	In single *TP53* ^mut^ with VAF < 50%, CK should be considered multi‐hit equivalent.
5	Confirmation of 17p13.1 deletion detected on karyotype by CNV analysis	Requires confirmation of loss of *TP53* locus detected on karyotype with an additional CNV method	Does not require confirmation of 17p13.1 deletion	Cases with single *TP53* ^mut^ (VAF < 50%) and 17p13.1 deletion on karyotype: CNV analysis verified LOH across the *TP53* locus in 94% cases [99]. Cases with single *TP53* ^mut^ (VAF < 50%) without 17p13.1 deletion on karyotype: CNV analysis identified LOH/cnLOH in 26.9% of evaluable cases, all of which harbored CK [99].	In single *TP53* ^mut^ with VAF < 50%, confirmation of 17p deletion with an additional CNV may not be necessary except complex rearrangements and structural changes
6	Morphology	Requires MDS diagnosis	Blasts < 5%: Cytopenia, does not require diagnostic dysplasia. Blast 5%–19%: Any cytopenia ≥ 20%/PEL: no requirement for cytopenia	—	Additional evidence needed
7	Prior cytotoxic therapies	Myeloid neoplasms post‐cytotoxic therapy (MN‐pCT), a part of “secondary myeloid neoplasms”	Diagnostic qualifier	In t‐MDS, survival of single‐hit *TP53* is comparable to multi‐hit loss (10.2 vs. 9.7 mo.; *p* = 0.59) [9].	Single‐hit t‐MDS should be included in *TP53* ^mut^ MN.

Abbreviations: AML—acute myeloid leukemia; AML‐MR—AML with myelodysplasia‐related; BM—bone marrow; cnLOH—copy neutral LOH; CNV—copy number variation analysis; LOH—loss‐of‐heterozygosity; MDS—myelodysplastic syndrome; MN‐pCT—myeloid neoplasms post‐cytotoxic therapy; mo.—months; PB—peripheral blood; PEL—pure erythroid leukemia; t‐MDS—therapy‐related myelodysplastic syndrome.

At the core of the above noted discrepancies is the ability to denote a neoplastic clone that completely lacks wild‐type TP53 function. Ideally, this would be established by demonstrating inactivation of both the alleles via ≥ 2 mutations or at ≥ 1 mutation with the deletion of the trans allele in the same cell. In the absence of routinely available single‐cell techniques to reliably establish biallelic inactivation, multi‐hit *TP53* inactivation is considered to be an acceptable surrogate. Urgent efforts are underway to provide additional clarifications in the matter and will help inform future iterations of the guidelines [9, 25, 26]. Below we review the emerging evidence that is expected to provide clarifications in the areas of discrepancy.

1. *TP53*‐mutated AML. Approximately 35%–40% of *TP53* ^mut^ MN are AML. A vast majority (94%) of these are discrepantly diagnosed between the ICC and WHO‐5 since ICC recognizes AML with mutated *TP53* ^mut^ VAF ≥ 10%, whereas a vast majority would instead be classified as AML, myelodysplasia‐related (AML‐MR) by WHO‐5 [101]. However, *TP53* ^mut^ AML showed a distinct genetic profile and significantly worse overall survival [99, 100]. Survival of *TP53* ^mut^ AML was significantly poor compared to *TP53* ^wt^ AML‐MR with myelodysplasia related (4.7 vs. 18.3 months; *p* < 0.0001) [99]. Collectively, these results support distinguishing *TP53* ^mut^ AML from other AML and incorporating in the *TP53* ^mut^ MN category [99, 100].
2. Variant allele frequency threshold. Another critical difference between the two classifications is the adoption of a VAF threshold of ≥ 10% in the ICC, but not WHO‐5. Specifically, ICC mandates *TP53* ^mut^ VAF ≥ 10% with an implicit rationale of distinguishing it from smaller *TP53* ^mut^ clones that may not be the pathologic driver. It is understood that the adopted VAF threshold is an empiric threshold to distinguish MN driven by *TP53* ^mut^ from those harboring *TP53* ^mut^. Approximately 10% of *TP53* ^mut^ MDS‐EB1, MDS‐EB2, and AML had *TP53* ^mut^ VAF < 10% and 55.6% of these cases harbored CK. Survival of MDS‐EB1, EB2 with VAF < 10% without CK was comparable to monoallelic MDS‐LB without CK (26.2 vs. 34.8 months; *p* = 0.44), whereas survival of MDS‐EB1, EB2 with VAF < 10% with CK was comparable to their counterparts with VAF ≥ 10% (5.6 vs. 6.3 months). Collectively, these results demonstrate that *TP53* ^mut^ MN cases with ≥ 5% blasts with CK should be included in *TP53* ^mut^ MN regardless of the VAF ≥ 10% or < 10% [99].
3. Interaction of the blast percentage categories with allelic status. The two classifications differ in their interpretation of the interaction between the blast percentage categories and allelic/hit status. The ICC prioritizes blast percentage in risk stratification, proposing 3 subcategories: multi‐hit MDS (0%–9% BM/PB blasts), MDS/AML (10%–19% BM/PB blasts), and AML (≥ 20% BM/PB blasts) regardless of allelic status. Consequently, single‐hit MDS (0%–9% BM/PB blasts) is excluded from the definition of *TP53* ^mut^ MN. On the other hand, WHO‐5 requires the demonstration of biallelic *TP53* inactivation throughout the spectrum of MDS (blasts 0%–19%), thus excluding monoallelic *TP53* ^mut^ MDS with 0%–19% blasts.

We have recently shown that the allelic status is of critical prognostic importance for cases with MDS‐low blasts (BM blast < 5% and blood blasts < 2%); but the prognosis of MDS‐EB1 (BM blasts 5%–9% and/or PB blast 2%–4%), MDS‐EB2 (BM blasts 10%–19% and/or PB blast 5%–19%), and AML (BM/PB blast ≥ 20%) is poor regardless of the allelic status. This is in agreement with another study demonstrating that MDS with blasts < 5% is a distinct subgroup compared to other categories. For example, only 24% of *TP53* ^mut^ MDS with < 5% harbored multi‐hit *TP53* inactivation compared to 67%, 91%, and 71% of cases with blasts 5%–9%, 10%–19%, and ≥ 20%, respectively [26]. Second, CK status predicted poor survival in MDS < 5% (hazard ratio, 5.2; *p* < 0.001) but not in the higher blast categories [26]. Combined, adopting WHO‐5 and ICC would underestimate the survival of single‐hit *TP53* ^mut^ MDS 5%–19% and 10%–19%, respectively.

The prevalence of cytogenetic abnormalities, including CK, is not homogeneously spread across all *TP53* ^mut^ MN. Instead, there is a complex interplay between CK, blast percentage, VAF, and allelic status (Figure 3C, Shah et al., *Blood Adv*, in print) [26, 57]. Given this complex interaction, a hierarchical prognostic model that simultaneously accounts for multiple factors is needed.

4 Complex karyotype is a practical surrogate of multi‐hit *TP53* inactivation. In the absence of comprehensive CNV analysis, CK (in the context of *TP53* ^mut^ VAF ≥ 10%) is considered a multi‐hit equivalent by ICC, but not by WHO‐5. We have reported that the survival of MDS cases with one *TP53* ^mut^ VAF < 50% with CK was comparable to those with 17p13.1 deletion on karyotype (10.4 vs. 11.0 months; *p* = 0.39) but was significantly poorer than those cases with monoallelic *TP53* ^mut^ without 17p loss or CK (10.4 vs. 33.4 months; *p* < 0.0001), indicating that for MDS cases with single *TP53* ^mut^ VAF < 50%, the presence of CK can be considered a practical surrogate for biallelic *TP53* inactivation.
5 Verification of 17p13.1 deletion detected by metaphase karyotype by copy number variation analysis. WHO‐5 mandates verification of 17p13.1 deletion detected by metaphase karyotype by an additional CNV method. In MDS with a single *TP53* ^mut^ with VAF < 50%, mere detection of 17p13.1 del on karyotype is not considered to be evidence of biallelic inactivation, while ICC does not mandate such verification.

Verification of loss across the *TP53* locus by CNV analysis is useful, especially in cases with complex structural rearrangements of 17p13.1 on karyotype. Second, metaphase karyotype can miss cryptic LOH/cnLOH.

Given the wide spectrum of instruments and personnel availability at different labs, an algorithmic approach that is highly dependent upon resources, turn‐around time, and institutional practices, timely CNV analysis is fraught with practical limitations: the majority of diagnostic laboratories cannot reliably analyze CNV using the routinely used NGS. SNP array and/or FISH studies would be pursued after *TP53* ^mut^ status is known. These sequential tests can substantially delay the risk stratification and management decisions.

This was illustrated in a recent international study of 603 MN harboring *TP53* ^mut^ MN, where ~20% had CNV analysis (Shah et al., under review). Of the evaluable cases with 17p13.1 deletion on karyotype, LOH across the *TP53* locus could be confirmed by CNV analysis in 94% of cases. Moreover, in cases without 17p13.1 deletion on karyotype, CNV analysis detected LOH/cnLOH across the *TP53* locus in 22.5% of cases. Importantly, all these cases harbored CK, and none of the cases without CK and 17p13.1 deletion on metaphase karyotype had LOH/cnLOH across the *TP53* locus. Furthermore, the survival of single *TP53* ^mut^ MDS with VAF < 50% and 17p13.1 del on metaphase karyotype was similar to cases with biallelic inactivation. Collectively, these results indicated that in clinical practice, 17p13.1 deletion on metaphase cytogenetics can be considered as evidence of the loss of the trans allele.

6 Morphological assessment. WHO‐5 requires that a case meets the MDS diagnostic criteria before it can be further classified as MDS with biallelic *TP53* inactivation (MDS‐bi*TP53*) [98]. On the other hand, ICC guidelines allow for the classification of multihit *TP53* ^mut^ cytopenic cases harboring < 5% blasts in the absence of dysplastic features as MDS with mutated *TP53*. This recommendation was based on a study that demonstrated that CCUS cases harboring *TP53* ^mut^ that lack sufficient dysplasia to diagnose MDS share biologic features, mutational patterns, and survival with *TP53* ^mut^ MDS [102]. Recent larger, population‐based studies did not confirm *TP53* ^mut^ as an independent adverse risk factor for survival [103, 104]. Finally, in a single institutional study of 29 *TP53* ^mut^ CCUS patients, only 3 (10.3%) progressed to MN, and the progression‐free and overall survival of *TP53* ^mut^ CCUS were comparable to *TP53* ^wt^ CCUS at up to 5 years [105]. Therefore, additional data is required to support future recommendations.
7 Impact of prior cytotoxic therapies. The two classifications differ in the interpretation of whether prior cytotoxic therapy modulates the risk‐stratification. ICC removed the subcategory of “therapy‐related myeloid neoplasms,” substituting it with diagnostic qualifiers instead [86]. The WHO‐5 has grouped t‐MN with secondary MN and renamed it as “myeloid neoplasm post‐cytotoxic therapy” [98]. In either cases, no distinctions were made between de novo and post‐cytotoxic therapy *TP53* ^mut^ MN. These recommendations were based on an international study of 229 t‐MDS, of which 18% (*n* = 41) harbored *TP53* ^mut^ that showed inferior survival of multi‐hit *TP53* ^mut^ t‐MDS compared to single‐hit *TP53* ^mut^ [24]. In contrast, analysis of a larger *TP53* ^mut^ t‐MN (*n* = 260, 34.2% *TP53* ^mut^) showed that unlike in de novo *TP53* ^mut^ MN, clinical features, structural chromosomal abnormalities, as well as co‐mutation pattern were comparable between single‐ and multi‐hit *TP53* ^mut^ t‐MN [9]. Critically, unlike de novo MDS [24], the incidence of AML progression and survival of single‐hit *TP53* ^mut^ t‐MDS were comparable to multi‐hit *TP53* ^mut^ t‐MDS.

These findings challenge the underlying assumption of the ICC classification that *TP53* ^mut^ MN—regardless of the underlying etiology—has similar genomic characteristics and outcomes and cautions against the underestimation of the poor prognosis of single‐hit *TP53* ^mut^ t‐MDS [9].

**FIGURE 4:**
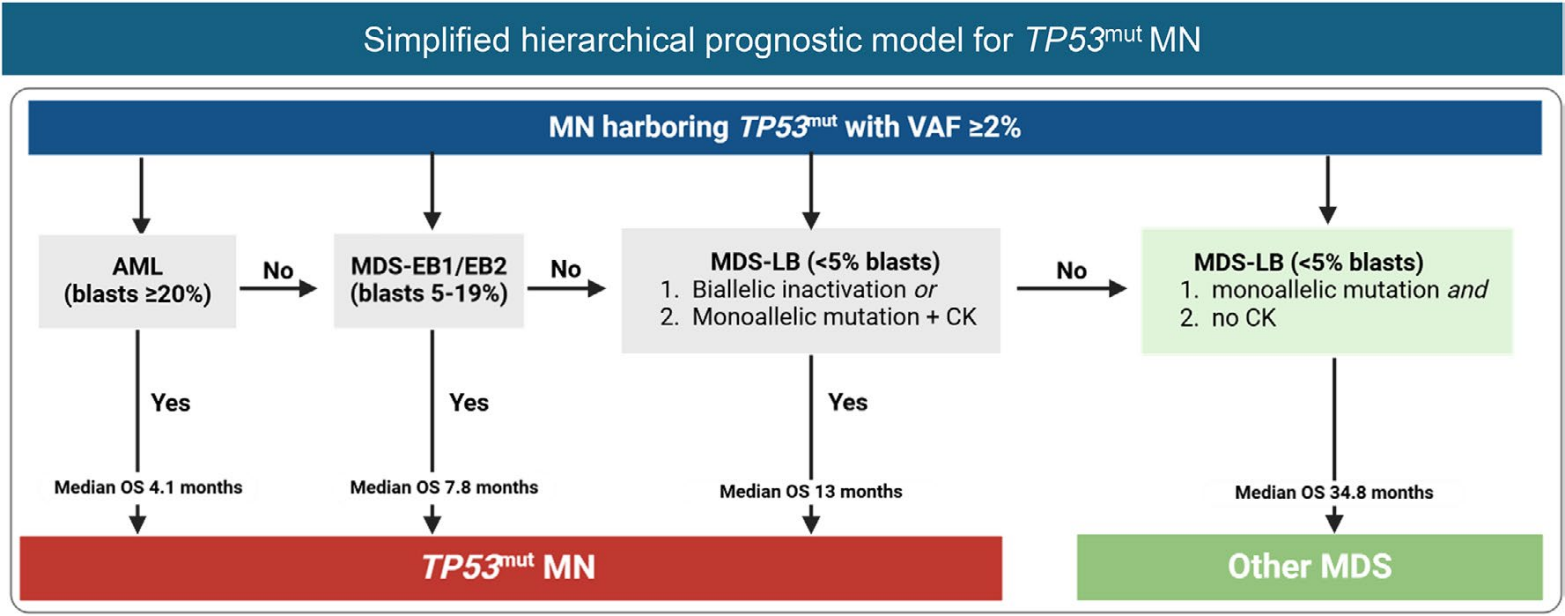
Evidence‐based hierarchical classification of *TP53*‐mutated (*TP53* ^mut^) myeloid neoplasms (MN). AML—acute myeloid leukemia; CK—complex karyotype; EB—excess blasts; LB—low (< 5% blasts); MDS—myelodysplastic syndrome; OS—overall survival (calculated from diagnosis); VAF—variant allele frequency. Adapted from Shah et al. (*Blood Adv*, in print).

Based on the evidence presented above [9, 26, 100], we propose a hierarchical model (Shah et al., *Blood Adv*, in print). Since *TP53* ^mut^ BM/PB ≥ 5% with VAF < 10% and no CK are relatively uncommon (4.2%), all *TP53* ^mut^ MN (VAF ≥ 2%) can be considered *TP53* ^mut^ MN. Accepting this stipulation, a simplified and clinically useful model is proposed (Figure 4) that acknowledges poor survival of 91.9% *TP53* ^mut^ MN.

## 6 Management of *TP53*‐Mutated Myeloid Neoplasms

Effective management of *TP53* ^mut^ MN continues to remain a long‐standing challenge, primarily due to its intrinsic chemorefractory nature [8, 9, 10]. Thus, despite its prevalence in cancer, TP53 remains undruggable. The notable progress made in other forms of MN has not benefited *TP53* ^mut^ MN and survival following the diagnosis remains < 1 year. *C*ommonly used strategies include HMA, intensive chemotherapies, and BCL‐2 inhibitor venetoclax. Initial studies of venetoclax—given high response rates—raised the hope that it would improve outcomes of *TP53* ^mut^ AML. However, with longer follow‐up, it is clear that venetoclax‐based regimens do not lead to a meaningful improvement in the survival of *TP53* ^mut^ AML [11, 12].

The lack of progress was evident in a recent meta‐analysis that observed comparable survival (6.5, 6.1, and 6.2 months) with intensive chemotherapy, HMA, and venetoclax plus HMA, respectively [106]. Disconcertingly, further development of several classes of drugs has been terminated due to lack of efficacy [13]. Below we summarize the published experience of commonly used management strategies.

1. Supportive care. Given age, comorbidities, concurrent cytopenia, and enrichment of t‐MN, 9.4%–21% of patients do not pursue disease‐modifying therapies, resorting to supportive care alone [9, 46]. Outcomes with a palliative care approach are poor as expected, with a median survival of 1–4 months [9, 46].
2. Intensive chemotherapy. Response to cytotoxic chemotherapies is highly dependent on the presence of intact p53 to enable the induction of apoptosis [107, 108]. Hence, *TP53* mutated MN respond poorly to chemotherapy. Historically, the combination of anthracyclines with cytarabine or a high‐dose cytarabine‐based regimens was used as the frontline therapy for patients deemed fit to receive intensive therapy [109]. The uptake of intensive chemotherapy is traditionally lower in *TP53* ^mut^ MN (14.6%–22.5%) [9, 46, 57], reflecting both patient and disease‐related factors: age, frailty, aggressiveness, and inherent chemorefractoriness that is characteristic of *TP53* ^mut^. Model response rate (20%–40%) and median survival (5–11 months) have been reported by multiple groups [8, 9, 57]. Interestingly, the presence of hotspot *TP53* ^mut^ was associated with an inferior response rate compared to those with non‐hotspot mutations (17.9% vs. 57.1%; *p* = 0.025) [46].

In a single‐institution study of 202 *TP53* ^mut^ AML, 22% received intermediate/high‐dose cytarabine‐based regimens. The response rate and survival in this cohort correlated with *TP53* ^mut^ VAF: those with VAF ≤ 40% had a median survival of 18.1 months compared to 5 months for patients with VAF > 40%. Moreover, the benefit of receiving a higher‐intensity therapy (compared to HMA) was limited to patients with VAF ≤ 40% [110]. Whether these findings represent a true therapeutic benefit of cytarabine‐based regimens or a representation of a younger cohort who underwent allogeneic transplant is unclear. Therefore, independent validation of this observation is awaited.

CPX‐351 is an encapsulated formulation of liposomal daunorubicin and cytarabine that preferentially delivers a synergistic 5:1 drug ratio into the leukemia cells, minimizing off‐target toxicities to the normal bone marrow cells. CPX‐351 is approved by the FDA for secondary AML and t‐AML—both of which are enriched for *TP53* ^mut^, though stratification based on *TP53* ^mut^ was not performed [111]. As with other modalities, composite complete responses were half in *TP53* ^mut^ compared to *TP53* ^wt^ AML (33% vs. 66%, *p* = 0.035) [112].

3 HMA. HMA such as 5‐azacitidine and decitabine represent one of the most prevalent strategies for MN including *TP53* ^mut^ MN. HMA were considered the preferred frontline therapy in elderly/unfit patients before the venetoclax era [113]. A retrospective analysis of the ASTRAL‐1 trial that included 17% *TP53* ^mut^ AML identified *TP53* ^mut^ as an “adverse‐risk feature” [114]. The poor outcome was confirmed in an independent retrospective analysis [115]. Welch et al. reported that 10‐day decitabine therapy ameliorated the adverse impact of *TP53* ^mut^ resulting in comparable survival of *TP53* ^mut^ and *TP53* ^wt^ (12.7 vs. 15.4 months, *p* = 0.79) [116]. However, a similar benefit could not be validated in another study that reported comparable response rate (40% vs. 43%, *p* = 0.78) and survival (6 vs. 5·5 months) with 10‐ vs. 5‐day decitabine regimen [117].
4 Venetoclax. Venetoclax, an orally available selective BCL‐2 inhibitor, has revolutionized the treatment for elderly AML patients or those ineligible for intensive induction. Pivotal studies VIALE‐A [118] and VIALE‐C [119] that studied venetoclax in combination with 5‐azacitidine and low‐dose cytarabine (LDAC), respectively, showed higher composite response rates (CR + CRi) with the addition of venetoclax (55.3% vs. 0% in VIALE‐A [118] and 18% vs. 0% in VIALE‐C) [119]. However, it was soon realized that decreased expression of BAX in *TP53*‐deficient AML cells contributed to their inherent resistance to BH3 mimetics [120]. In addition, monocytic differentiation and downregulation of HLA‐DR and CD34 were suggested as a potential mechanisms of acquired resistance [121].

Upon longer follow‐up, it is now quite clear that venetoclax‐based regimens do not improve outcomes for this subset [122, 123, 124]. In a recent study of 301 newly diagnosed AML patients treated with venetoclax plus HMA, multi‐hit *TP53* ^mut^ was associated with lower CR/CRi compared to single‐hit *TP53* ^mut^ or *TP53* ^wt^ AML (38% vs. 63% vs. 67%). *TP53* ^mut^ also conferred an inferior relapse‐free survival (7.9 vs. 19.3 months) and overall survival (5.9 vs. 16.6 months) compared to *TP53* ^wt^ AML [125]. Another pooled analysis of 279 patients treated on the pivotal venetoclax plus azacitidine studies confirmed *TP53* ^mut^ as an independent risk factor for survival (median 5.5 months). Combined, these observations have led to *TP53* ^mut^ as the defining feature of the “adverse‐risk” category in the ELN risk classification for patients receiving less‐intensive therapies [126].

Given the FDA approval, the majority of the evidence for the efficacy of venetoclax‐based regimen is limited to AML. Recently, venetoclax combinations have been tried in high‐risk MDS including *TP53* ^mut^ MDS [127]. In a recent phase 1b study of venetoclax with azacitidine for treatment‐naive HR‐MDS (NCT02942290), 20 (18.7%) of 107 patients harbored *TP53* ^mut^. *TP53* ^mut^ cases had a comparable rate of complete remission to the entire cohort (25% vs. 29%), though survival was numerically inferior (11.2 vs. 26 months). Longer follow‐up and larger studies will be needed to ascertain these findings.

5 Allogeneic hematopoietic cell transplant. AlloHCT is the only modality with curative potential for patients with *TP53* ^mut^ MN. A retrospective analysis of the Blood and Marrow Transplant Clinical Trials Network 1102 study that randomized high‐risk MDS patients, including *TP53* ^mut^ MDS, based on donor availability showed significantly improved long‐term survival in patients undergoing alloHCT compared to non‐transplant approaches (3‐year OS 23% vs. 11%, *p* = 0.04). Therefore, alloHCT remains the “gold‐standard” for all eligible patients [128].

**FIGURE 5:**
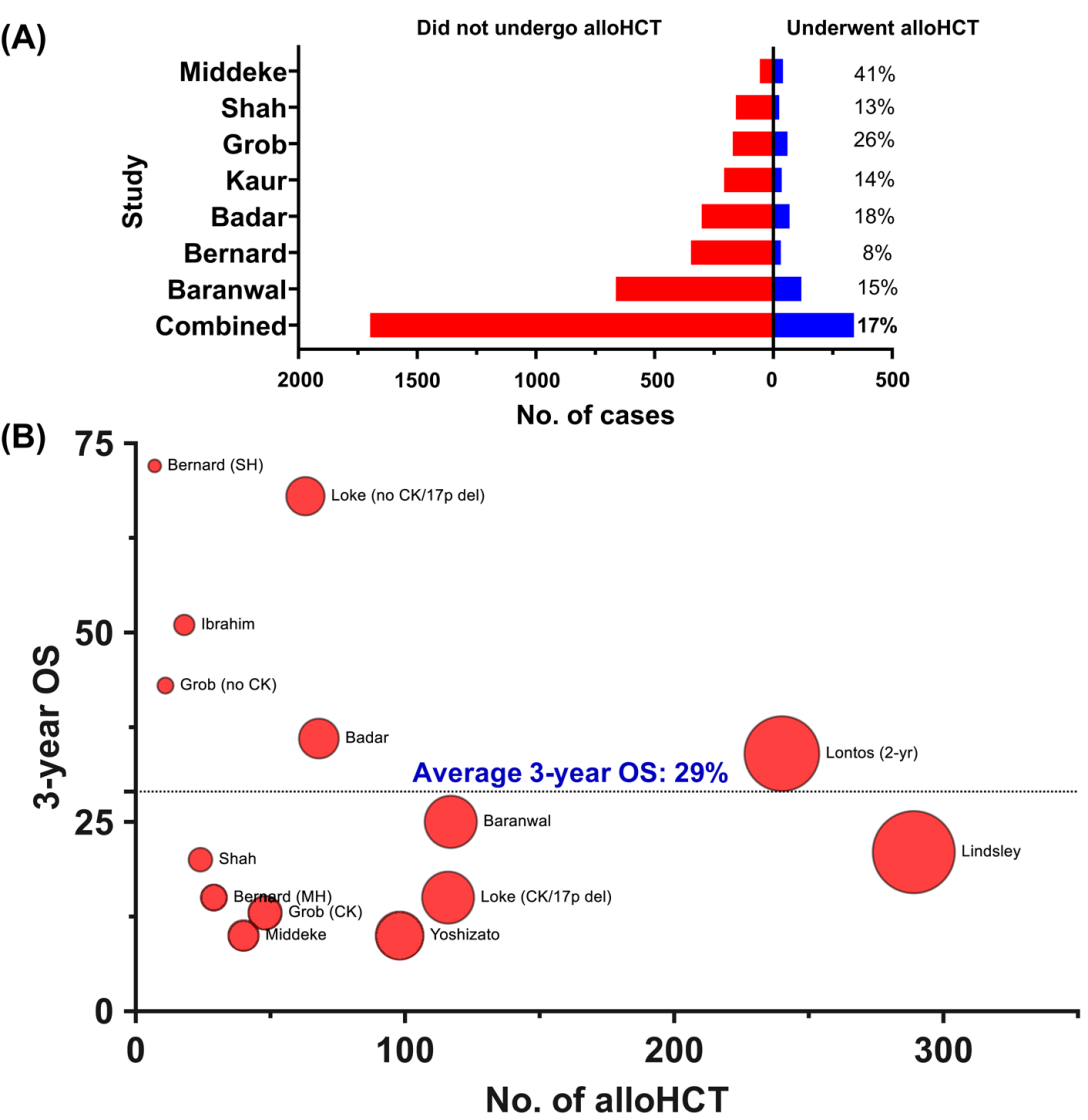
Outcomes following allogeneic hematopoietic cell transplant (alloHCT) for myeloid neoplasms harboring *TP53* mutations (*TP53* ^mut^). (A) Utilization of alloHCT remains low in *TP53* ^mut^ MN. (B) Three‐year overall survival following alloHCT for *TP53* ^mut^ MN across published studies. Studies include: Badar et al. [131], Baranwal et al. (in print), Bernard et al. [24], Grob et al. [8], Ibrahim et al. [132], Kaur et al. [46], Lindsley et al. [2], Loke et al. [133], Lontos et al.** [134], Middeke et al. [135], Shah et al. [57], and Yoshizato et al. [136] 17p del—17p deletion; CK—complex karyotype; MH—multi‐hit; SH—single‐hit. **Overall survival at 2 years.

On the other hand, given the resource‐intense nature as well as high morbidity and mortality, there is an active debate around the applicability of *TP53* ^mut^ MN [129, 130]. Historically, only 7%–18% of *TP53* ^mut^ MN undergo alloHCT (Figure 5A) [9, 46, 111, 131]. In the absence of systematic studies, the reasons for the low utilization are speculative and include older age, frailty, comorbidities, concurrent malignancies, inability to achieve the desired pre‐alloHCT response, and high post‐alloHCT relapse risk.

Three‐year survival is 10%–15% in most published studies regardless of the MDS or AML phenotype (Figure 5B). A recent international multicenter study (7 transplant centers in USA and Australia, Baranwal et al., in print) of 134 *TP53* ^mut^ MN patients who underwent alloHCT confirmed poor survival in the contemporary era: median post‐HCT survival was 1.03 years and OS at 1‐, 2‐, and 3‐years was 51.4%, 35.1%, and 25.1%, respectively. Multihit *TP53* inactivation was associated with significantly shorter 3‐year survival compared to those with non‐multihit *TP53* inactivation (16.9% vs. 54.9%, *p* = 0.002). Interestingly, non‐DBD *TP53* ^mut^ only and *DNMT3A* co‐mutation were associated with 3.4‐ and 2.6‐fold lower relapse‐free survival (RFS). These results, for the first time, suggested the mutational and co‐mutational patterns associated with post‐HCT outcomes. These results require validation in an independent cohort.

Similar findings are reported in a single‐center analysis of 240 MN cohort [134]. Survival was comparable between *TP53* ^mut^ MDS and AML, and 2‐year OS for the entire cohort was 34%. Hierarchical analysis identified 3 groups: best survival was in cases with *TP53* ^mut^ VAF < 50% that did not harbor CK/deletion 5q/7q, followed by *TP53* ^mut^ VAF < 50% that harbored CK/deletion 5q/7q, and the worst being the cases with *TP53* ^mut^ VAF ≥ 50% with CK/deletion 5q/7q (2‐year OS of 60%, 22%, and 3% respectively).

Post‐transplant mortality and morbidity are driven by the high relapse rate. Therefore, multiple studies have attempted to identify factors associated with lower post‐alloHCT relapse. Unfortunately, no clear picture emerges, though the younger recipient age [2], *TP53* ^mut^ VAF < 40% [110], single‐hit *TP53* ^mut^ (Baranwal et al., in print), absence of CK [133], undergoing alloHCT in 1st remission [131], achieving CR at day +100 [46, 131], and the development of moderate to severe GVHD [46, 131] were associated with favorable survival. Therefore, there is an active debate if (a) the disease status at alloHCT and (b) the choice of conditioning intensity impact post‐alloHCT survival.

As to the former, in *TP53* ^mut^ EB‐2/AML patients treated with intensive chemotherapy, achieving minimal residual disease (MRD) negativity was not associated with longer post‐alloHCT survival [8]. In another multicenter study, achieving MRD negative CR before alloHCT was not associated with improved relapse‐free or overall survival [131]. On the other hand, in a study by Hunter et al. [137], of the 16 *TP53* ^mut^ MN who underwent alloHCT, 7 achieved a clearance of *TP53* ^mut^ (by NGS) pre‐alloHCT and had a numerical trend towards improved survival (25.2 vs. 11.7 months). Interestingly, patients with pre‐alloHCT *TP53* ^mut^ clearance benefited from alloHCT over continued HMA (25.2 vs. 7.7 months); whereas those with clonal persistence had survival comparable between the two approaches (11.7 vs. 7.7 months). If validated, these studies can be the first step in identifying suitable candidates.

Second, the choice of optimal conditioning is far from certain: while some studies suggested a reduced risk of relapse using myeloablative conditioning (MAC), most do not demonstrate a survival benefit [2, 131, 138]. For example, in a CIBMTR study of *TP53* ^mut^ MDS, both the risk of relapse and survival were comparable between MAC and reduced‐intensity conditioning (RIC) [2]. This was recently confirmed by another multi‐institutional study [131]. In contrast, others have shown a reduced risk of relapse but a higher risk of non‐relapse mortality with MAC, culminating still in a survival comparable to RIC regimens [138, 139].

Finally, two studies suggest an advantage of melphalan‐based conditioning. In a single‐institution study of t‐MDS patients undergoing alloHCT predominantly using melphalan‐based conditioning showed higher RFS and OS were shown compared to other studies, though the impact of melphalan could not be isolated [132]. In the multicenter study discussed above, the inclusion of melphalan was associated with improved RFS (HR 0.52, *p* = 0.005). The discrepancies above may—at least in part—be explained as the benefit of melphalan‐based conditioning (almost exclusively used in the RIC context) was limited to cases with < 5% blasts pre‐alloHCT (Baranwal et al., *Blood Adv*, in print).

### 6.1 Immunotherapies and Novel Targets

#### 6.1.1 Immunotherapy

Immunotherapy has taken center stage in oncology over the last decade. Immunotherapy agents rely on the innate ability of the immune system to detect and eliminate tumor cells. Checkpoint inhibitors function by blocking inhibitory co‐receptors on T‐cells, including programmed cell death (PD)‐1, programmed cell death ligand (PD‐L)‐1, and cytotoxic T‐lymphocyte antigen (CTLA)‐4 inhibitors. These proteins are accessories to immune evasion by tumor cells, and the expression of these receptors and their ligands is augmented in myeloid malignancies, suggesting a possible resistance mechanism to conventional therapies [140, 141]. As discussed above, *TP53* ^mut^ MN have a distinct immune *milieu*—raising the possibility that immune‐based therapies can be exploited in the treatment of *TP53*‐mutated AML. Current approaches under study include ICI, bispecific and dual‐antigen–receptor targeting antibodies, chimeric antigen receptor (CAR) T‐cell therapy, and newer targets such as T‐cell immunoglobulin and mucin domain (TIM)‐3 inhibitors. The stimulator of interferon genes are some novel treatment modalities under this domain [142].

- ICI. Contrary to solid malignancies and some hematological malignancies, *TP53* ^mut^ MN are modestly sensitive to ICI when combined with HMA or intensive chemotherapy. In a phase 2 study, nivolumab was used in combination with 5‐azacitidine in relapsed/refractory AML, 16 of whom harbored *TP53* ^mut^. The overall response rate for *TP53* ^mut^ AML was 19% (3/16 patients) [41]. Similarly, another phase II trial showed that pembrolizumab after high‐dose cytarabine was safe in 37 patients with R/R AML (overall response rate 46%, complete remission 38%, median overall survival 11.1 months). Two of the 5 (40%) patients with *TP53* ^mut^ in this cohort achieved complete remission [42]. Then, in a phase II trial of upfront nivolumab with idarubicin and cytarabine in 44 treatment‐naïve patients with high‐risk MDS/AML, including 8 with *TP53* ^mut^ MDS, the CR/CRi rate was 78% [43] Prospective, randomized studies are needed to assess if patients with *TP53* ^mut^ AML have better outcomes with ICI combinations than with conventional chemotherapies alone. Designing these trials will need to take a broader view as most patients will be planned to undergo alloHCT, potentially raising the concern for exacerbating graft‐vs.‐host disease [143].
- Sabatolimab is a monoclonal antibody targeting TIM‐3. In a phase Ib, multi‐arm, open‐label, multicenter study for AML, high/very high‐risk MDS, and chronic myelomonocytic leukemia, 14 evaluable patients with *TP53* ^mut^, the overall response rate was 71.4%, complete remission was seen in 4 (28.6%) and the median duration of response was 21.5 months [144]. STIMULUS‐MDS‐1 [145] was a multicenter, randomized, double‐blind, placebo‐controlled, phase II study in newly diagnosed high‐risk MDS including *TP53* ^mut^ MDS. Of 186 patients screened, 36% of the sabatolimab and 33% of the placebo cohort harbored *TP53* ^mut^. While response rates or survival were not stratified by *TP53* ^mut^ status, the primary endpoints were not met. Complete remission rate (22% vs. 18%, *p* = 0.77) and progression‐free survival (11.1 vs. 8.5 months, *p* = 0.1) were comparable in the sabatolimab and placebo groups. Ultimately, the phase III study (STIMULUS‐MDS‐2) failed to meet the primary endpoint of improved overall survival—resulting in the sponsor announcing discontinuation of the sabatolimab program [45].
- Cluster of differentiation (CD)‐47 is widely expressed across various cell types that interacts with signal‐regulatory protein (SIRP)‐α on phagocytic cells, delivering an anti‐phagocytic “don't‐eat‐me” signal. Its overexpression in cancer cells is hypothesized to counteract the pro‐phagocytic signals, avoiding phagocytosis. Magrolimab, a humanized immunoglobulin G4 anti‐CD47 monoclonal antibody, inhibits the CD47‐SIRPα interaction, enhancing cancer cell phagocytosis. In combination with 5‐azacitidine that upregulates calreticulin on AML cells, increasing the “eat me” signal. Based on the in vitro and in vivo preclinical data, a phase I study of magrolimab monotherapy was well tolerated in relapsed/refractory AML (NCT02678338, CAMELLIA study) [146] demonstrated satisfactory safety. Phase Ib (5F9005, NCT03248479) study combining magrolimab with azacitidine in newly diagnosed AML who were ineligible for intensive chemotherapy. Of 87 patients, 82.8% harbored *TP53* ^mut^ (median VAF 61%, range: 9.8–98.7). After a median of 4 (range, 1–39) cycles of therapy, complete remission rate was 31.9% in *TP53* ^mut^ AML. The median overall survival of *TP53* ^mut^ and *TP53* ^wt^ AML were 9.8 and 18.9 months, respectively. The median duration of response was 7.7 months. Among 14 *TP53* ^mut^ patients who achieved measurable residual disease (MRD)‐negative responses, median survival was 14.5 months compared to 7.5 months who remained MRD‐positive [147]. Subsequent randomized study of *TP53* ^mut^ AML patients who were ineligible for intensive chemotherapy were treated with magrolimab plus AZA vs. investigator choice (ENHANCE‐2) and AZA+ VEN+ magrolimab vs. AZA+VEN [148]. However, magrolimab trials have been discontinued due to futility based on planned analysis [149]. Similarly, trials using other anti‐CD47 monoclonal antibody (evorpacept, NCT04417517 and NCT04755244) have been terminated as the combination “did not substantially improve upon the historical activity of azacitidine alone.” Currently, there is one open trial of anti‐CD47 antibody with HMA in AML and high‐risk MDS (NCT06008405, Table 4), demonstrating continued interest in this target.
- Bispecific antibodies, bispecific T‐cell engagers, and dual affinity retargeting antibodies have been under exploration for the management of *TP53* ^mut^ AML. Flotetuzumab, a CD123xCD3‐targeting dual affinity retargeting antibody that works by enhancing the formation of an immunologic synapse between cytotoxic T‐ and AML cells independent of the major histocompatibility complex (MHC) pathway, has shown promising efficacy in *TP53* ^mut^ (complete remission 47%, median survival 10.3 months) [150]. Another bispecific antibody against CD123xCD3 (APVO436‐5001) has also shown efficacy and safety in an early‐phase clinical trial [151].
- Chimeric antigen T‐cell receptor therapy: Compared to other hematological malignancies, the development of CAR‐T therapy in MN has been confronted by toxicities including myelosuppression and poor CAR T‐cell persistence [152]. Despite that, multiple trials targeting established (CD33, CD123) and novel (CLL‐1, CD371) and combination targets are being investigated (Table 4).

**TABLE 4:** Select ongoing clinical trials for *TP53‐*mutated myeloid neoplasms.

NCT identifier	Title	Status	Condition	Intervention(s)	Phase
NCT06778187	Oral‐ATO for TP53‐mutated Myeloid Malignancies	Not yet Recruiting	AML, MDS	Oral arsenic trioxide	2
NCT04277442	Testing Nivolumab in Combination with Decitabine and Venetoclax in Patients with Newly Diagnosed TP53 Gene Mutated Acute Myeloid Leukemia	Active, not Recruiting	AML	Decitabine, nivolumab, venetoclax	1
NCT06456463	A Study of Tagraxofusp in Combination with Venetoclax and Azacitidine in Adults with Untreated CD123+ Acute Myeloid Leukemia Who Cannot Undergo Intensive Chemotherapy	Recruiting	AML	Tagraxofusp, venetoclax, 5‐azacitidine	2
NCT05396859	Entrectinib in Combination with ASTX727 for the Treatment of Relapsed/Refractory TP53 Mutated Acute Myeloid Leukemia	Recruiting	AML	Decitabine, cedazuridine, entrectinib	1
NCT06549790	Study of NMS‐03597812 in Adult Patients with Relapsed/Refractory Acute Myeloid Leukemia	Recruiting	AML	NMS‐03597812	1
NCT03772925	Pevonedistat and Belinostat in Treating Patients with Relapsed or Refractory Acute Myeloid Leukemia or Myelodysplastic Syndrome	Active, not Recruiting	AML, MDS	Belinostat, pevonedistat	1
NCT02665065	Study of Iomab‐B vs. Conventional Care in Older Subjects with Active, Relapsed or Refractory Acute Myeloid Leukemia	Active, not Recruiting	AML	Iomab‐B	3
NCT04358393	A Study of APG‐115 Alone or Combined with Azacitidine in Patients With AML, CMML, or MDS	Recruiting	AML, MDS	APG‐115, 5‐azacitidine	1/2
NCT06514261	Testing the Combination of an Anti‐Cancer Drug, Iadademstat, With Other Anti‐Cancer Drugs (Venetoclax and Azacitidine) for Treating AML	Recruiting	AML	Azacitidine, iadademstat, venetoclax	1
NCT04477291	A Study of CG‐806 in Patients with Relapsed or Refractory AML or Higher‐Risk MDS	Active, not Recruiting	AML, MDS	CG‐806	1
NCT03560882	A Pilot Trial of Atorvastatin in Tumor Protein 53 (p53)—Mutant and p53 Wild‐Type Malignancies	Active, not Recruiting	AML, MDS	Atorvastatin	1
NCT03850574	Clinical Trial to Evaluate the Safety, Tolerability, Pharmacokinetics and Pharmacodynamics of Tuspetinib (HM43239) in Patients with Relapsed or Refractory Acute Myeloid Leukemia	Recruiting	AML, MDS	Tuspetinib, venetoclax, 5‐azacitidine	1/2
NCT06008405	Clinical Trial Evaluating the Safety of the TQB2928 Injection Combination Therapy	Recruiting	AML, MDS	TQB2928 (anti‐CD47)	1
NCT06130579	Interferon‐alpha for TP53 Myeloid Malignancy Post Allo‐HSCT	Recruiting	AML, MDS	IFN‐alpha	2
NCT04219163	Chimeric Antigen Receptor T‐cells for the Treatment of AML Expressing CLL‐1 Antigen	Recruiting	AML	CLL‐1 CAR	1
NCT05672147	CD33‐CAR T Cell Therapy for the Treatment of Recurrent or Refractory Acute Myeloid Leukemia	Recruiting	AML	CD33 CAR	1
NCT04923919	Clinical Study of Chimeric Antigen Receptor T Lymphocytes (CAR‐T) in the Treatment of Myeloid Leukemia	Recruiting	AML	CLL‐1 CAR	1
NCT06326021	Optimised CD33 (FL‐33) CAR T Therapy for Refractory/Relapsed Acute Myeloid Leukaemia	Recruiting	AML	FL‐33 CAR	1
NCT05017883	TAA05 Cell Injection in the Treatment of Recurrent/Refractory Acute Myeloid Leukemia	Recruiting	AML	TAA05 CAR	
NCT05984199	Donor‐Derived Anti‐CD33 CAR T Cell Therapy (VCAR33) in Patients with Relapsed or Refractory AML After Allogeneic Hematopoietic Cell Transplant	Recruiting	AML	VCAR33	1/2
NCT06765876	CART123 T Cells in Relapsed or Refractory CD123+ Hematologic Malignancies: A Dose Escalation Phase I Trial	Recruiting	AML	CAR123, autologous	1
NCT06197672	Chimeric Antigen Receptor T Cell Redirected to Target CD4 Positive Relapsed Refractory Acute Myeloid Leukemia (AML) As a Bridge to Allogeneic Stem Cell Transplant	Recruiting	AML	CD4, autologous	1
NCT05457010	Phase I Study of Cell Therapies for the Treatment of Patients with Relapsed or Refractory AML or High‐risk MDS	Recruiting	AML	SPRX002/ARC‐T cells	1
NCT06125652	Administration of Anti Tim‐3/CD123 CAR‐T Cell Therapy in Relapsed and Refractory Acute Myeloid Leukemia (rr/AML)	Recruiting	AML	TIM‐3/CD123 CAR	1/2
NCT03971799	Study of Anti‐CD33 Chimeric Antigen Receptor‐Expressing T Cells (CD33CART) in Children and Young Adults with Relapsed/Refractory Acute Myeloid Leukemia	Recruiting	AML	CD33 autologous/allogeneic CAR	1/2
NCT05488132	Administration of Anti‐siglec‐6 CAR‐T Cell Therapy in Relapsed and Refractory Acute Myeloid Leukemia (rr/AML)	Recruiting	AML	siglec‐6 CAR	1/2
NCT06609928	FH‐FOLR1 Chimeric Antigen Receptor T Cell Therapy for Treating Pediatric Patients with Relapsed or Refractory Acute Myeloid Leukemia	Recruiting	AML	FOLR1 CAR	1
NCT05949125	Phase 1 Study of Allo‐RevCAR01‐T‐CD123 in Patients with Selected CD123 Positive Hematologic Malignancies	Recruiting	AML	CD123, allogeneic CAR	1
NCT04230265	Phase 1 Study of UniCAR02‐T‐CD123 in Patients with Selected CD123 Positive Hematologic Malignancies	Recruiting	AML	CD123 CAR	1
NCT04265963	CD123‐Targeted CAR‐T Cell Therapy for Relapsed/Refractory Acute Myeloid Leukemia	Recruiting	AML	CD123 CAR	1/2
NCT06762132	A Clinical Study to Explore the Safety and Efficacy of CD33 CAR‐T Cell in Relapsed/Refractory Acute Myeloid Leukemia	Recruiting	AML	CD33 CAR	1
NCT04272125	Safety and Efficacy of CD123‐Targeted CAR‐T Therapy for Relapsed/Refractory Acute Myeloid Leukemia	Recruiting	AML	CD123 CAR	1/2
NCT04803929	Clinical Study of Anti‐ILT3 CAR‐T Therapy for R/R AML(M4/M5)	Recruiting	AML	ILT3 CAR	1
NCT03190278	Study Evaluating Safety and Efficacy of UCART123v1.2 in Patients with Relapsed/Refractory Acute Myeloid Leukemia	Recruiting	AML	UCART123v1.2	1
NCT06017258	A Study of CD371‐YSNVZIL‐18 CAR T Cells in People with Acute Myeloid Leukemia	Recruiting	AML	CD371‐specific/YSNVz/I‐18 CAR	1
NCT05748197	A Study of ADCLEC.syn1 in People with Acute Myeloid Leukemia	Recruiting	AML	ADCLEC.syn1	1
NCT06642025	EX02 CAR‐T Cells for Relapsed and Refractory Acute Myeloid Leukemia	Recruiting	AML	EX02 CAR	1
NCT06709131	A Clinical Study to Explore the Safety and Efficacy of CT0991 in Relapsed/Refractory Acute Myeloid Leukemia	Recruiting	AML	CT0991 CAR	1
NCT06420063	Sequential CAR‐T Cells Targeting CD33/CD123 in Patients with Acute Myelocytic Leukemia AML	Recruiting	AML	CD33/CD123 CAR	1/2
NCT03291444	CAR‐T Cells Combined with Peptide Specific Dendritic Cell in Relapsed/Refractory Leukemia/MDS	Recruiting	AML	CAR	1
NCT05995041	Universal CAR‐T Cells Targeting AML	Recruiting	AML	CLL‐1, CD33, CD38 and/or CD123 CAR	1
NCT05945849	CD33KO‐HSPC Infusion Followed by CART‐33 Infusion(s) for Refractory/Relapsed AML	Recruiting	AML	CD33 CAR	1
NCT06128044	CRISPR‐Edited Allogeneic Anti‐CLL‐1 CAR‐T Cell Therapy in Patients with Relapsed/Refractory Acute Myeloid Leukemia	Recruiting	AML	CLL‐1 CAR	1
NCT04662294	CD 70 CAR T for Patients with CD70 Positive Malignant Hematologic Diseases	Recruiting	AML	CD70 CAR	1
NCT05105152	PLAT‐08: a Study of SC‐DARIC33 CAR T Cells in Pediatric and Young Adults with Relapsed or Refractory CD33+ AML	Recruiting	AML	CD33 CAR	1
NCT04318678	CD123‐Directed Autologous T‐Cell Therapy for Acute Myelogenous Leukemia (CATCHAML)	Recruiting	AML	CD123 CAR	1
NCT05377827	Dose‐Escalation and Dose‐Expansion Study to Evaluate the Safety and Tolerability of Anti‐CD7 Allogeneic CAR T‐Cells (WU‐CART‐007) in Patients with CD7+ Hematologic Malignancies	Recruiting	AML	CD7 CAR	1

*Note*: List retrieved from clinicaltrials.gov on February 22, 2025.

Abbreviations: AML—acute myeloid leukemia; CAR—chimeric antigen T‐cell receptor therapy; MDS—myelodysplastic syndrome.

#### 6.1.2 Novel Targeted Therapies

- TP53‐activator: Eprenetapopt (APR‐246) is a novel, first‐in‐class, small molecule that restores wild‐type p53 functions in TP53‐mutant cells. It was evaluated in a phase Ib/II study. Of 55 patients (40 MDS, 11 AML, 4 MDS/MPN) with at least one *TP53* mutation^mut^ treated, the overall response rate was 71%, with 44% achieving CR. Among MDS patients, 50% achieved CR, and 58% had a cytogenetic response. The overall response rate and complete remission rate in AML were 64% and 36%, respectively. Interestingly, responders had a significant reduction in *TP53* ^mut^ VAF, with 38% achieving complete molecular remission. Median overall survival was 10.8 months. Thirty‐five percent of patients underwent allogeneic hematopoietic stem‐cell transplant, with a median overall survival of 14.7 months [153]. Based on these exciting results, a combination phase I multicenter study with HMA and venetoclax was conducted. Forty‐nine patients were enrolled. The combination was well tolerated, and the overall response with eprenetapopt and venetoclax with azacytidine was 38% [154]. Despite these encouraging results, further development of the drug is unfortunately halted due to futility following a phase III study [155].
- Splenic tyrosine kinase inhibitors: Splenic tyrosine kinase (Syk) is overexpressed in AML, and the overactivity is associated with poor prognosis. Entospletinib is a Syk inhibitor that was evaluated in combination with decitabine for *TP53* ^mut^ or CK AML in the BEAT AML Study. The drug combination showed modest activity—leading to discontinuation due to futility [156].
- ROS1 inhibitor: Various malignancies, including AML, demonstrate aberrant expression of the *ROS* proto‐oncogene 1 receptor tyrosine kinase, making it a viable target for anticancer therapies. TP53‐deficient cells show sustained sensitivity to the ROS1 inhibitor entrecitinib [157]. Based on these results, a phase I study is evaluating entrecitinib, in combination with a hypomethylating agent, as a treatment for relapsed/refractory *TP53* ^mut^ AML (Table 4).
- Nutlin analogs: Given the central role of *MDM2* as a negative regulator of TP53, small molecular inhibitors of MDM2, such as nutlin analogs, have been explored for *TP53* ^mut^ MN. In the Phase 1b trial of idasanutlin plus venetoclax in relapsed/refractory AML, an overall response rate was seen in 3 of 10 *TP53* ^mut^ (no complete remissions), and the median duration of response and survival were merely 2.3 and 3.67 months. *TP53* ^mut^ was subclonal in responders. At discontinuation, 25 *TP53* ^mut^ were noted in 12 patients—of which 22 were pre‐existing [158]. The phase 3 MIRROS trial was a multicenter, randomized, double‐blind, phase 3 study of the MDM2 antagonist idasanutlin plus cytarabine in relapsed/refractory AML. The study enrolled patients regardless of *TP53* ^mut^ status with the rationale that some *TP53* ^mut^ could retain wild‐type TP53 function. Approximately 15% of patients enrolled harbored *TP53* ^mut^. Endpoints, including overall survival (median, 8.3 vs. 9.1 months *p* = 0.58), complete remission (20.3% vs. 17.1%), and overall response rate (38.8% vs. 22.0%) were all comparable between idasanutlin plus cytarabine compared to placebo plus cytarabine [159]. Overall, consistent with the mechanism, these results suggest the possibility that MDM2 inhibitors may not be effective in *TP53* ^mut^ MN.

The above encounters highlight the unparallelled therapeutic challenges presented by *TP53* ^mut^ MN. We conclude that the development of effective therapies for *TP53* ^mut^ MN is an urgent unmet clinical need.

## 7 Future Directions

An unwavering interest of scientists and clinicians alike in all aspects of *TP53* ^mut^ MN is a testament to the enormity of the challenge. The review above highlights advances made in our understanding of the disease, but also substantial challenges that persist. It is hoped that the recognition as a separate entity will stimulate research, facilitate clinical trial enrollment, and fuel drug discovery. To that end, we propose the following as the areas of the highest priority.

Not all *TP53* ^mut^ CH progress to MN, and the typical latency seen is > 5 years. Documented stability of the *TP53* ^mut^ clone without leukemic transformation and the dramatic contrast between the outcomes of *TP53* ^mut^ CH/CCUS and *TP53* ^mut^ MN, collectively highlight the critical need to characterize the factors associated with leukemic transformation. Specifically, the availability of biomarker(s) will help identify high‐risk patients and will guide surveillance strategy. This is even more critical for patients with known *TP53* ^mut^ CH in need of informed decision to undergo cytotoxic therapy for unrelated malignancy. Ultimately, it is hoped that early interventions such as pre‐emptive alloHCT before the acquisition of biallelic *TP53* inactivation and leukemic transformation would improve outcomes.

Second, future iterations of the *TP53* ^mut^ MN classification are expected to incorporate the emerging evidence. The current diagnostic criteria aim to overcome the technical limitation of assigning the biallelic loss confidently, necessitating the use of surrogates such as “presumed biallelic” or “multihit equivalent.” In the future, incorporation of WES/WGS or preferentially single‐cell studies will help localize the cell‐of‐origin and localize biallelic *TP53* alterations in leukemic cells. While such technology is available, a globally adopted classification must balance feasibility (availability, personnel need, turn‐around‐time, and cost) with accuracy.

Third, with near‐universal use of NGS and progressively increasing sensitivity, clinicians are expected to encounter small *TP53* ^mut^ clones of uncertain significance. Studies reporting outcomes used variable thresholds of *TP53* ^mut^ VAF (ranging from 1% to 20%) [2, 6, 8, 10]. Therefore, additional clarity is needed to determine if an optimal *TP53* ^mut^ VAF reliably distinguishes CH/CCUS from MN and those with poor survival.

Finally, at the peril of stating the obvious, ultimate progress will be measured by offering safe, effective, and durable treatments. The collective approach would include early institution of therapy and achieving higher response rates—potentially increasing the patients eligible for alloHCT.

## Ethics Statement

The authors have nothing to report.

## Conflicts of Interest

M.V.S. receives research funding to the institution from Astellas Pharma, AbbVie, KURA Oncology, Inc., BMS/Celgene, and Marker Therapeutics. D.K.H. is a member of the board of directors or advisory committees of AbbVie and Novartis.

## Data Availability

The authors have nothing to report.
